# Hybridization of Wide-Angle X-ray and Neutron Diffraction Techniques in the Crystal Structure Analyses of Synthetic Polymers

**DOI:** 10.3390/polym15020465

**Published:** 2023-01-16

**Authors:** Kohji Tashiro, Katsuhiro Kusaka, Hiroko Yamamoto, Takaaki Hosoya, Shuji Okada, Takashi Ohhara

**Affiliations:** 1 Aichi Synchrotron Radiation Center, Knowledge Hub Aichi, Minami-Yamaguchi, Seto 489-0965, Japan; 2 Frontier Research Center for Applied Atomic Sciences, Ibaraki University, Shirakata 162-1, Tokai, Naka 319-1106, Japan; 3 Department of Future-Industry Oriented Basic Science and Materials, Toyota Technological Institute, Tempaku, Nagoya 468-8511, Japan; 4 College of Engineering, Ibaraki University, 4-12-1 Nakanarusawa, Hitachi 316-8511, Japan; 5 Graduate School of Science and Engineering, Yamagata University, Yonezawa 992-8510, Japan; 6 J-PARC Center, Japan Atomic Energy Agency, 2-4 Shirakata, Tokai, Naka 319-1195, Japan

**Keywords:** wide-angle X-ray diffraction, wide-angle neutron diffraction, crystal structures, crystalline polymers, X-N method, bonded electron density distribution, phase transition

## Abstract

The development in the crystal structure analysis of synthetic polymers using the hybridized combination of wide-angle X-ray and neutron diffraction (WAXD and WAND, respectively) techniques has been reviewed with many case studies performed by the authors. At first, the technical development was reviewed, in which the usage of high-energy synchrotron X-ray source was emphasized for increasing the total number of the observable diffraction peaks, and several examples were introduced. Secondly, the usage of the WAND method was introduced, in which the successful extraction of hydrogen atomic positions was described. The third example is to show the importance for the hybrid combination of these two diffraction methods. The quantitative WAXD data analysis gave the crystal structures of at-poly(vinyl alcohol) (at-PVA) and at-PVA-iodine complex. However, the thus-proposed structure models were found not to reproduce the observed WAND data very much. The reason came from the remarkable difference in the atomic scattering powers of the constituting atomic species between WAXD and WAND phenomena. The introduction of statistical disorder solved this serious problem, which reproduced both of the observed WAXD and WAND data consistently. The more systematic combination of WAXD and WAND methods, or the so-called X-N method, was applied also to the quantitative evaluation of the bonded electron density distribution along the skeletal chains, where the results about polydiacetylene single crystals were presented as the first successful study. Finally, the application of WAND technique in the trace of structural changes induced under the application of external stress or temperature was described. The future perspective is described for the development of structural science of synthetic polymers on the basis of the combined WAXD/WAND techniques.

## 1. Introduction

Since polymer science was started in early 1900s, X-ray crystal structure analysis has been playing an important role in the study of the structure-property relationship of synthetic polymers viewed from the atomistic level [1,2,3,4,5]. Unfortunately, in contrast to the remarkable progress of the X-ray structure analysis of single crystals of low-molecular-weight compounds, the developing pace of the crystal structure analysis of polymer materials is quite slow. There are still many unsolved problems even for such representative polymer substances as polyethylene, isotactic polypropylene, atactic poly(vinyl alcohol), poly(ethylene terephthalate), and so on.

The analytical techniques of crystal structures based on the X-ray diffraction theory have been developed stepwise by introducing the various ideas: (i) how to collect the larger number of the X-ray diffraction peaks, (ii) how to analyze the position and integrated intensity of the broad and overlapping peaks in a quantitative manner, (iii) how to find the initial models consistent with the thus-analyzed data, and (iv) how to refine the models so that the observed data can be reproduced not only for the integrated intensity but also for the one-dimensional and two-dimensional diffraction patterns [5]. By finding the various technical methods to solve these problems, for the first time, it may be said that we obtain *the most plausible structure model*. The authors dare to use such ironic words as “the most plausible model”. Even after the completion of the data analysis using the above-mentioned techniques; unfortunately, we might have still serious problems about the thus-derived structure models. The reasons come from such a real situation that the total number of the observed diffraction peaks is limited to only several tens, at best, for many kinds of synthetic polymers. In order to find the correct answer about the structure, in general, the total number of the observed diffraction peaks must be 3–4 times larger than the total number of structural parameters to be determined (the positional coordinates and thermal parameters of the atoms). For example, a unit cell is assumed to consist of *M* crystallographically asymmetric units, which are related to each other by the space group symmetries. The *N* atoms are assumed to be contained in one asymmetric unit. The total number of atoms in the unit cell is *MN*. However, it is enough to find the coordinates and thermal parameters of the *N* atoms in one asymmetric unit, since the information of the other units is obtained by the symmetry relations. The independent parameters to be determined are the (*x*, *y*, *z*) coordinates and one global isotropic temperature factor *B* of the *N* atoms, then the total number of parameters should be 3*N* + 1 + 1, where the last “1” comes from the scale factor to adjust the total sum of the diffraction intensities between the observed and calculated values. If *N* = 10, the total number of parameters is 32. Then, we have to collect 32 × 3~32 × 4 = 96~128 diffraction peaks. The number of the actually observed diffraction peaks is only 20, for example. In order to escape from such a physically difficult situation in the X-ray structure analysis of synthetic polymers, people have been seeking the various ideas. One successful technique is to use an X-ray source of higher energy or an X-ray beam of shorter wavelength to collect more number of diffraction signals. For example, the usage of synchrotron X-ray beam of wavelength 0.33 Å, instead of the 1.54 Å beam often used in the laboratory X-ray equipment, can increase the observable number of diffraction spots by one order, as will be shown concretely in a later section. This technique can overcome one of the requirements necessary for the accurate structure analysis. The remarkable progress in the X-ray detectors cannot be forgotten. The development of so-called two-dimensional photon counter makes it possible to collect the digitized diffraction data at a higher signal-to-noise ratio in a wide dynamic range of 1–10^6^ photons in a short time, which can be treated quantitatively by using the various excellent computer programs to collect an accurate set of diffraction data (the indices and integrated intensities of the separated peak components).

The neutron diffraction method is one of the most useful techniques complementary to the X-ray method. We can use the bulky sample and analyze the diffraction data by using essentially the same concept of the reciprocal lattice. Since the atomic scattering powers are different between the X-ray and neutron beams depending on the atomic species, the structure information derived from these two analyses can mutually check the reasonableness of the derived structural model, as will be described in a later section. For example, the X-ray wave is diffracted by H atoms. However, the scattering power of H atom is quite low compared with the other heavier atomic species (C, O, etc.) since the scattered X-ray intensity is proportional to total number of electrons. Contrarily, the D atom can scatter the *coherent* neutron wave with a larger scattering amplitude comparable to that of C atom. This situation may make it possible to determine the H (D) atomic positions in the unit cell by analyzing the neutron diffraction data collected for the deuterated sample. The information of H atomic positions is indispensable in the quantitative prediction of the anisotropic physical property of the crystal phase, because the intermolecular non-bonded H…H interactions, in addition to the conformation of the skeletal chains, have the most serious effect on the anisotropic property. More generally, as mentioned, the atomic scattering power of neutron is not necessarily simply related to the total number of electrons, but it is sensitively dependent on the atomic elements. For example, the heavy iodine atom gives quite weak neutron scattering compared with the C and D atoms. As a result, the structure model derived by the detailed X-ray diffraction data analysis might be overridden by the neutron diffraction analysis. The concrete examples will be shown in a later section. In this way, the organized combination of X-ray and neutron diffraction techniques may be one of the most powerful methods to determine the crystal structure and the structure–property relation of synthetic polymers with high reliability.

Unfortunately, however, the literature challenging this hard theme has been limited in the long history of polymer science. The threshold to approach the neutron diffraction study of synthetic polymers is high compared with that of the X-ray method in the various points. For example, in the neutron experiment, the H atom gives overwhelmingly large *incoherent* background but the D atom gives rather strong *coherent* signal, so we need to prepare the deuterated samples to collect the coherently diffracted data necessary for the structure analysis. Collaboration with the chemists is desirable to prepare the deuterated polymer samples [6]. Another high barrier is how to increase the power of incident neutron beam. At present, the neutron source, which is obtained by a nuclear fission of ^235^U in the reactor or by the spallation of heavy atom by the bombardment of high-energy protons, is not very powerful compared with the X-ray beam (for example, synchrotron X-ray beam). Therefore, the neutron diffraction experiment must be performed by irradiating the beam on a sample of a large volume (a few mm–cm size) for a long time (several hours to days). Because of these serious situations, the neutron diffraction experiment is still minor in the structure analysis of synthetic polymers, although this situation is now changing gradually through the development of neutron facility.

Of course, the structure analysis is not carried out only by the X-ray and neutron diffraction techniques. The utilization of such other analytical methods as vibrational spectroscopy, solid-state NMR spectroscopy, electron diffraction technique, computer simulation method, and so on may be able to fill the unknown or ambiguous parts of the structural information derived by the X-ray and/or neutron analyses [5]. These analytical techniques have their own excellent characters and give us the unexpected ideas or knowledge useful for the forward advancement of the structure analysis. However, in the case of electron diffraction method, for example, it requires an ultrathin film to avoid the strong absorption of electron beam by the sample. Moreover, the range of the observable diffraction angle is limited due to the limited spatial area. The multiple scattering effect of the electron signals inside a single crystal may modify the relative intensity among the diffraction signals and make the situation appreciably complicated [5,7]. Recently, the so-called cryo-type transmission electron microscope has become popular for obtaining the direct images of giant protein molecules [8]. In a near future, the application to the synthetic polymer substance might be tried. At present, however, the electron diffraction method has serious limitations in the structural analysis of synthetic polymers.

In this way, we have described the reasons why the combination of X-ray and neutron diffraction methods is indispensable for the reliable crystal structure analysis of synthetic polymers. In the present paper, we will review some of our research accumulated by using the synchrotron high-energy X-ray beam and the wide-angle neutron diffraction system. The thus-obtained structure information has made it possible to understand the physical properties of the synthetic polymers from the atomic level. In an extreme case, the study of the bonded electron density distribution along the polymer chain has been performed successfully, which might be one excellent test case for the future progress in the structural science of synthetic polymers from the electronic level [5].

## 2. Experimental Systems

### 2.1. X-ray Diffraction System

As mentioned above, the accurate structure analysis of a polymer crystal needs as many diffraction peaks as possible, which must be collected in a wide diffraction angle and at the high signal-to-noise ratio. It is much better to use the X-ray beam of as short a wavelength as possible. The practically useful X-ray beam is that generated from the synchrotron light source. The wavelength of 0.33 Å is used in our studies. The reason why we need to use the X-ray beam of such a shorter wavelength comes from the principle of X-ray diffraction by the crystal lattice [5]. As seen in the textbook, the so-called Bragg’s law of reflection is given as

2*d_hkl_* sin(θ) = λ (1)

where *d_hkl_* is the stacking spacing of the *hkl* planes, and 2θ is the Bragg diffraction angle. Equation (1) is equivalent to the following equation:

***k*** = (***k***_s_ − ***k***_i_)/λ = ***p***_hkl_* (2)

**Figure 1:**
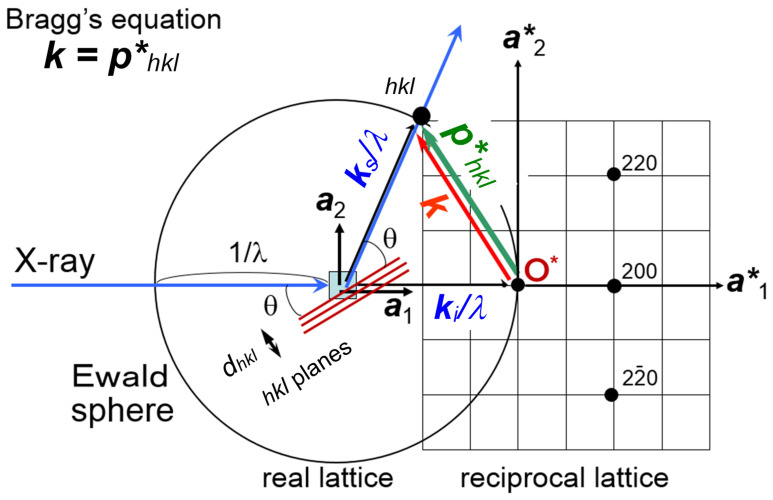
Ewald sphere and Bragg’s reflection. The crossing point of a scattering vector ***k*** with a reciprocal lattice point *hkl* causes the Bragg’s reflection on the *hkl* planes of the spacing *d_hkl_* in the real lattice. The shorter wavelength of an incident X-ray beam gives the larger Ewald sphere, increasing the crossing points.

**Figure 2:**
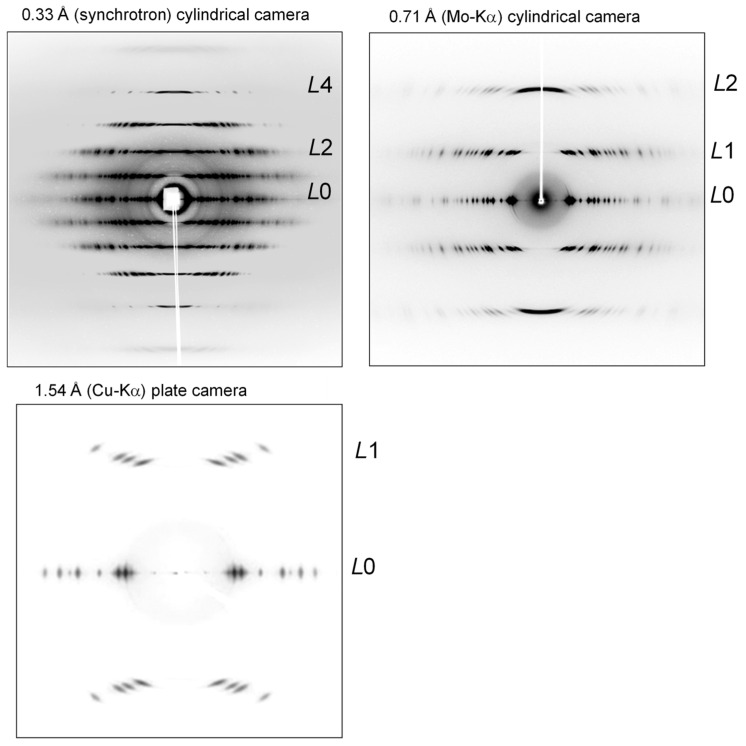
2D wide-angle X-ray diffraction patterns measured for an ultra-drawn polyethylene sample using the X-ray beam of the various wavelengths. *L* indicates the layer line [9].

The ***k***_i_ and ***k***_s_ are the unit vectors representing the directions of the incident and scattered X-ray beams, respectively; |***k***_i_| = |***k***_s_| = 1. These two vectors are different in direction by the angle 2θ. The ***k*** is the scattering vector and it has a magnitude |***k***| = (2/λ)sin(θ). The ***p***_hkl_* is the reciprocal lattice vector with the magnitude 1/*d_hkl_*. The Equation (2) indicates that the Bragg reflection should occur when the scattering vector ***k*** is equal to ***p***_hkl_*. This geometrical relation can be understood by calling the concept of Ewald sphere. The Ewald sphere of radius 1/λ is set at the sample position. The origin of the reciprocal lattice space is coincident with the edge of the sphere (Figure 1). It is said that the Bragg reflection from the *hkl* planes occurs when the vector ***p***_hkl_* crosses the Ewald sphere. The diffracted X-ray signal goes toward the direction connecting the sample and the crossing point at the Bragg 2θ angle. The total number of the reciprocal lattice points crossing the sphere is equal to the number of the observable diffraction spots. Therefore, the total number of the observed diffractions increases if the radius of the sphere becomes larger. In other words, the larger number of the observed diffraction peaks can be collected by using the X-ray beam of a shorter wavelength. Figure 2 shows one typical example demonstrating this situation clearly [9]. The sample is highly oriented Polyethylene fiber. Compared with the data collected using an X-ray beam of a longer wavelength (Cu-Kα, 1.54 Å, and Mo-Kα, 0.71 Å), the usage of synchrotron X-ray beam of 0.33 Å gave a one-order-larger number of the observed peaks. Another important point is to use the detector of a high sensitivity covering a wide intensity range (1–10^6^ photons). The digitized photon-counters are now quite popular in the X-ray scattering experiments. However, these detectors are not necessarily fitted to the experiment of the polymer materials. The size of one pixel of the detector is desired to be as small as possible. Of course, the higher spatial resolution is better since the profile (or shape) of the observed diffraction peak is important in the structure analysis of the polymer material, different from the small and sharp peaks obtained for the single crystals of small molecules. The usage of an imaging plate is useful for the polymer materials since the pixel size is appreciably smaller than that of the popular photon counting detector.

### 2.2. Neutron Diffraction Systems

The wide-angle neutron diffraction data are measured mainly by two methods. One is to use the monochromatized neutron beam originated from the atomic reactor, and another one is to use the neutron pulses generated by the so-called spallation method.

#### 2.2.1. Monochromatic Neutron Beam

**Figure 3:**
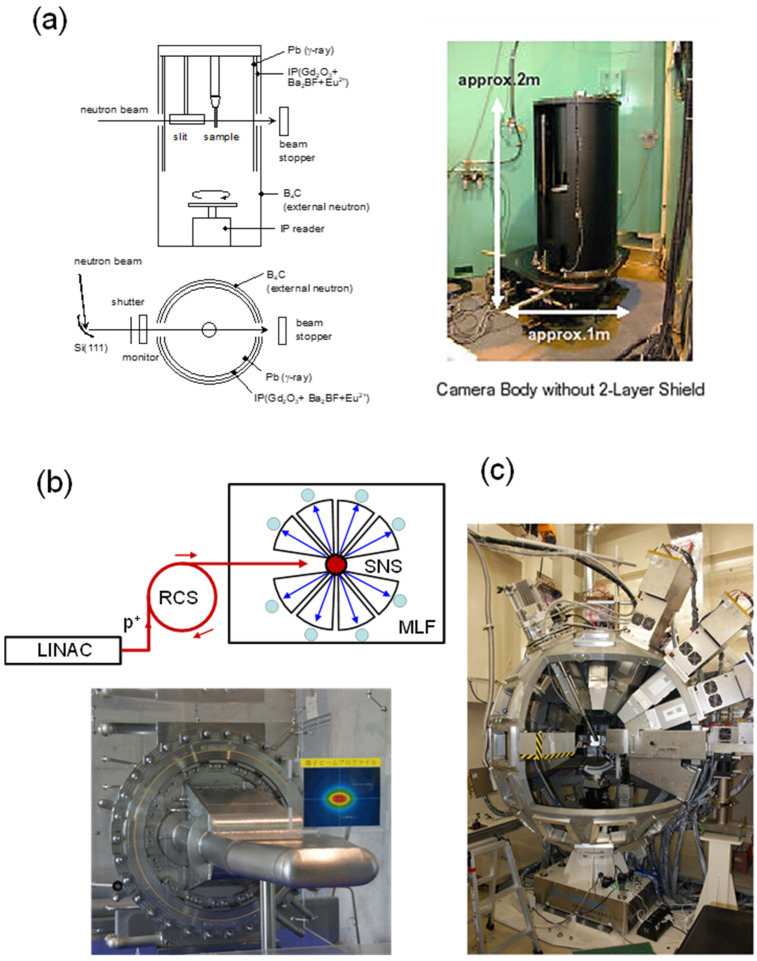
(**a**) A BIX-3 system set in the JRR-3, (**b**) a neutron pulse generation system in the MLF (Materials and Life Science Experimental Facility) of J-PARC by utilizing a proton (p^+^)-bombardment-induced spallation of mercury, and (**c**) an iBIX system set in the beam line 03 of MLF. In (**b**), LINAC: linear accelerator, RCS: rapid-cycling synchrotron, and SNS: spallation neutron source with mercury target vessel (the picture).

The neutron is created by a nuclear fission of ^235^U species. The most representative neutron facility in Japan is JRR-3 of the JAEA (Japan Atomic Energy Association [10]). The neutron beam of a unique wavelength is extracted as an incident beam using such a monochromator as silicon single crystal (Figure 3a). The thus-obtained neutron beam is not very powerful. Therefore, a highly sensitive detector must be used. The 2D imaging plate specialized for the neutron signal collection is useful, which is installed in the BIX-3 system of the JRR-3 [11]. The sample must have a large volume of about 5 mm × 5 mm × 5 mm. A long exposure time of several tens of hours is needed to obtain the diffraction data at relatively high signal-to-noise ratio.

#### 2.2.2. Neutron Pulses

The proton particles accelerated almost closely to a light velocity are bombarded to a liquid Hg tank, from which the strong neutron beams are scattered through the nuclear fission. The radially emitted neutron beams are led toward the individual experimental hatches (Figure 3b). Since the protons are injected discontinuously at a constant period, the neutron signals are pulses, which consists of the various neuron waves of the different wavelengths or the different velocities (*v*). The neutron signals scattered by the sample reach the detector at the different timing. This is called a TOF (time-of-flight) method. Using the de Broglei’s equation (λ = *h/*(*m*_o_*v*)), the Bragg equation is expressed as

2*d* sin(θ) = λ = *h/*(*m*_o_*v*) = *t*(*h*/*m*_o_)/*L* (3)

where *m*_o_ is a neutron mass, *h* is a Planck constant, *L* is a flight distance of the neutron, and *t* is a flight time (*L* = *vt*). One detector set at a fixed 2θ direction collects the neutron signals of the various *d* spacings at the different timings. The thus-accumulated 3D data as a function of (*x*, *y*, *t*) coordinates are converted to a series of the 2D diffraction patterns of (1/*d*_x_, 1/*d*_y_) coordinates at the different *t*s.

#### 2.2.3. Sample Preparations for WAND Experiments

The polymer samples for the X-ray diffraction measurements should be as highly crystalline and highly oriented as possible in order to obtain the good diffraction patterns of many mutually separated peaks. The samples necessary for the WAND experiments must be the deuterated polymer species. Moreover, their volumes must be large to obtain the signals of high signal-to-noise (S/N) ratio. The reasons for the usage of deuterated samples are understood from Table 1.

**Figure 4:**
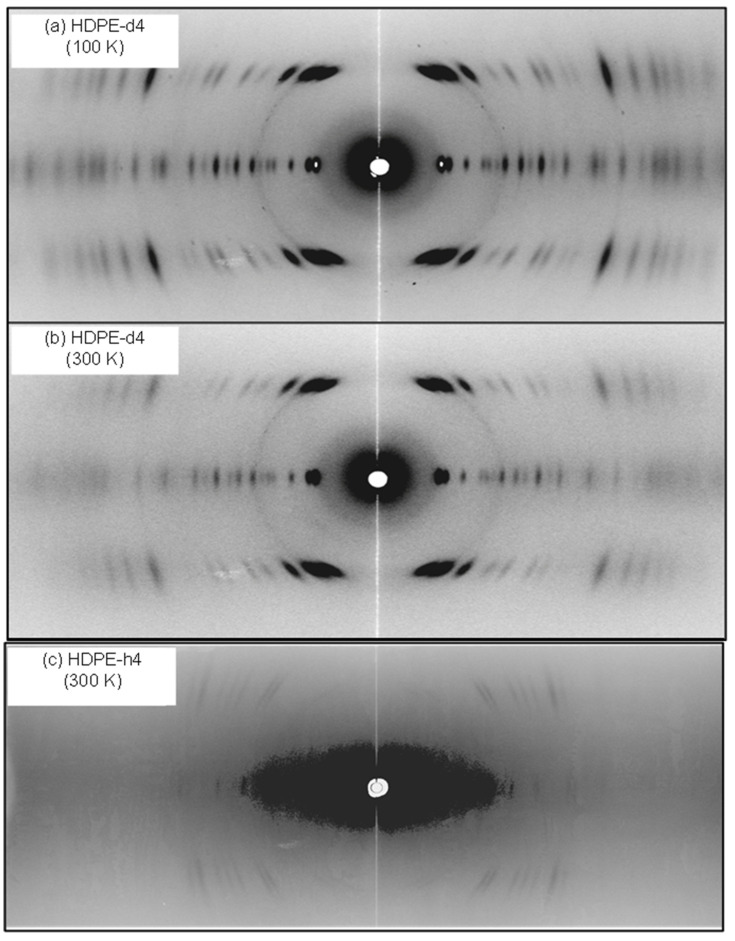
2D wide-angle neutron diffraction patterns measured for uniaxially oriented polyethylene samples (HDPE-d4 at 100 K and 300 K and HDPE-h4 at 300 K).

The X-ray scattering power is stronger in the order of the total number of electrons surrounding the nucleus. In contrast, the light H atom strongly scatters the incoherent neutron signals, which are observed as the strong background to cover the weaker coherent signals. On the other hand, the incoherent scattering from the D atom is overwhelmingly smaller. The D atom can scatter the coherent neutron signals relatively strongly, and the scattering power (cross-sectional area) is almost comparable to that of C atom. In fact, as seen in Figure 4, the hydrogenated high-density polyethylene (HDPE-h4, -[CH_2_CH_2_]_n_-) and the deuterated polyethylene (HDPE-d4, -[CD_2_CD_2_]_n_-) samples give the remarkably different diffraction patterns. The HDPE-d4 sample shows the clear diffraction pattern with weak background, while the PE-h4 sample exhibits the appreciably strong background and the quite weak coherent Bragg diffraction peaks. In addition, as mentioned already, the sample size must be large, about 5 mm diameter and 5 mm length. Such a large sample is difficult to prepare, and a bundle of the highly drawn rods was used as a sample in our experiments.

## 3. Several Examples of X-ray Crystal Structure Analysis of Polymers

To demonstrate the usefulness of the WAXD data analysis in the structure analysis of synthetic polymers, we will show here several examples of the crystal structures of biodegradable polymers.

### 3.1. PLLA α Form

**Figure 5:**
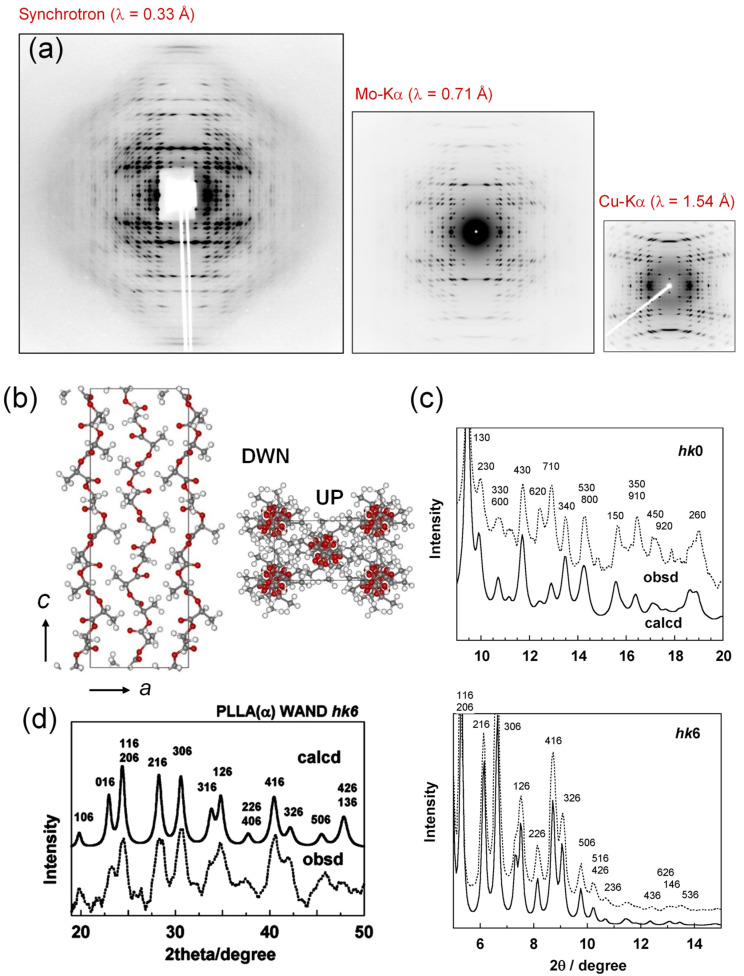
(**a**) 2D WAXD patterns of an ultra-drawn PLLA (α form) measured by the X-ray beams of various wavelengths, (**b**) the crystal structure of PLLA α form, (**c**) the comparison between the observed and calculated X-ray diffraction profiles along the equatorial (*hk*0) and the 6^th^ layer line (*hk*6), and (**d**) the comparison of the WAND profile (*hk*6) [12].

The high-energy synchrotron X-ray beam (of a short wavelength) gave us the beautiful 2D diffraction pattern of the uniaxially-oriented poly(L-lactic acid) (PLLA, -[CHCH_3_-OCO]_n_-) α form. By using about 700 diffraction peaks, the accurate crystal structure was derived as shown in Figure 5 [12]. This structure satisfied both of the observed X-ray and neutron diffraction data quite consistently. The molecular chains take approximately the 10/3 helical conformation, that is, the 3-turn helix consisting of 10 monomeric units in the repeating period along the chain axis. In more crystallographically strict expression, these chains do not possess any symmetry, slightly deformed from the regular helix. The ultimate Young’s modulus along the chain axis and the 3-dimensional elastic constants matrix were predicted by the lattice dynamical calculation based on the thus-determined crystal structure [13].

### 3.2. PLLA/PDLA Stereocomplex

**Figure 6:**
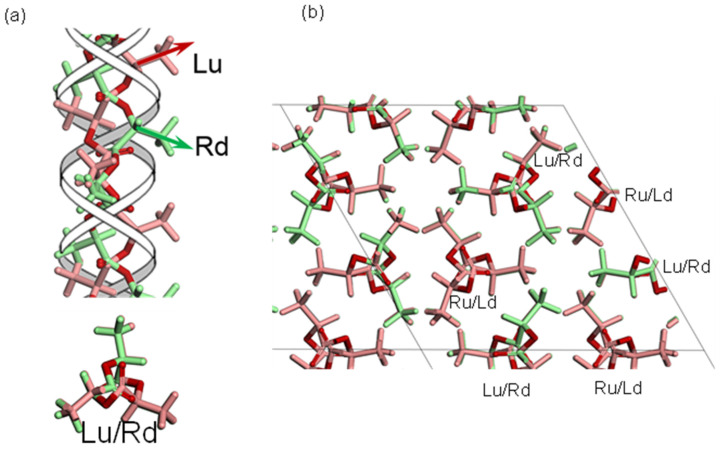
Crystal structure of PLLA/PDLA stereocomplex (PLLA/PDLA = 50/50) with the space group *P*3. (**a**) Left-upward (Lu) (3/1) helical chain and right-downward (Rd) (3/1) helical chain are located on the same lattice site at 50% probability, whose projected views along the chain axis give the same form. (**b**) The Lu/Rd chain and Ru/Ld chain are located at the adjacent sites, and these pairs are connected in the trigonal unit cell by the 3-fold rotation axes [15]. An ideal and regular packing structure of Ru and Lu chains is made by the space group symmetry *R*3*c* [16], which cannot cover all of the cases of L/D = 30/70~70/30 [15].

The blend samples between the optically enantiomeric PLLA and PDLA (poly-D-lactic acid) chains at the 3/7–7/3 molar ratio were found to produce the so-called stereocomplex in the solid-state after cooled from the melt [14]. The detailed X-ray data analysis gave the crystal structure shown in Figure 6 [15]. Both of the PLLA and PDLA chains take the 3/1 helices, but their handedness is opposite. So far, the structure of the space group *R*3*c* had been utilized popularly for the stereocomplex, in which the pairs of PDLA and PLLA chains are packed regularly and alternately side by side under the 3_1_ helical symmetric constrains [16]. However, the *R*3*c* structure model requires the serious constraints on both the chain conformation and the chain packing mode: only the upward (or downward) helices of PLLA and PDLA chains are packed in the unit cell at the ratio of 1:1 only. The various experiments suggested that the stereocomplex is formed in a wide range of PLLA/PDLA ratio of 3/7 to 7/3. The circular dichroic IR spectral data support also this point [17]. The newly proposed crystal structure is shown in Figure 6, which can satisfy the various experimental data consistently [15]. The space group is P3, lower than the *R*3*c* model. The pairs of PLLA and PDLA chain stems are packed in the trigonal cell under the 3-fold-rotation symmetry relation. Let us focus on one lattice site. For example, in the stereocomplex formed from the PDLA 70%/PLLA 30% mixture, one lattice site is occupied by the upward PDLA chain at 70% probability and the downward PLLA chain at 30% probability. Another site adjacent to this site is occupied by the downward PDLA chain of 70% probability and the upward PLLA chain of 30% probability. The shapes of the upward PLLA and the downward PDLA chains are the same as each other when viewed along the chain axis. The statistical coexistence of the upward and downward chains allows the formation of the thin lamellar structure with the folded chain stems, which are realized as the spherulites and detected as the long period in the small-angle X-ray scattering pattern.

### 3.3. Poly(3-Hydroxybutyrate)

**Figure 7:**
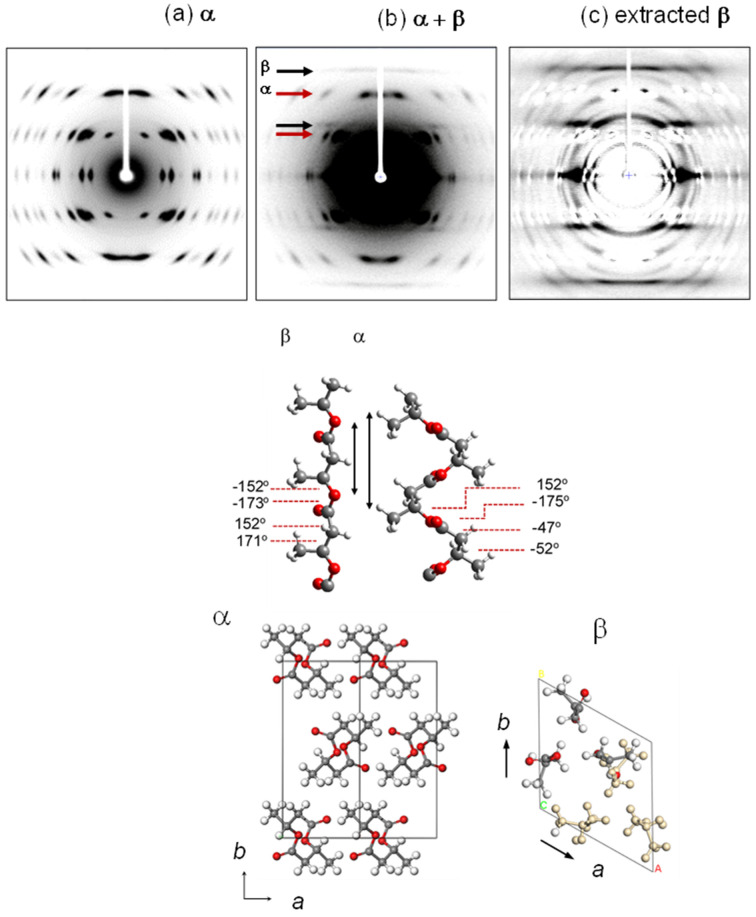
(upper) 2D X-ray diffraction patterns of the uniaxially-oriented PHB samples: (**a**) the pure α form, (**b**) the mixture of the α and β forms, and (**c**) the β form pattern obtained by the subtraction (**a**) from (**b**). (lower) The chain conformations and crystal structures of the α and β forms [20,23].

Poly(3-hydroxybutyrate) (PHB, -[CH_2_CHCH_3_-COO]_n_-) shows the crystal modifications of the α and β forms [18]. The α form is obtained when the sample is cooled from the melt. The α form takes the orthorhombic unit cell of *P*2_1_2_1_2_1_ symmetry, as shown in Figure 7 [19]. The 2_1_ helical chains of TTGG conformation are connected by the 2_1_ screw symmetries. The detailed X-ray data analysis indicated a little short non-bonded H…O distance between the CH_3_ and O=C groups belonging to the neighboring chains [20]. This suggests the existence of some unusual interactions between the H (CH_3_) and O (C=O) atoms. The anomalous interactions are consistent with such a spectroscopic observation that one particular IR band of the asymmetric CH_3_ stretching modes locates at a remarkably high frequency position compared with those observed for the other substances. Sato et al. proposed the formation of C-H…O=C intermolecular hydrogen bonds as the secondary interactions in addition to the normal van der Waals interactions [21]. This secondary interaction makes the free rotation of CH_3_ groups difficult to occur, as known from the experimental observation that the CH_3_ torsional motion starts at around 140 K, which is appreciably higher than the temperature 100 K observed for the CH_3_ rotation of isotactic polypropylene [20,22].

**Figure 8:**
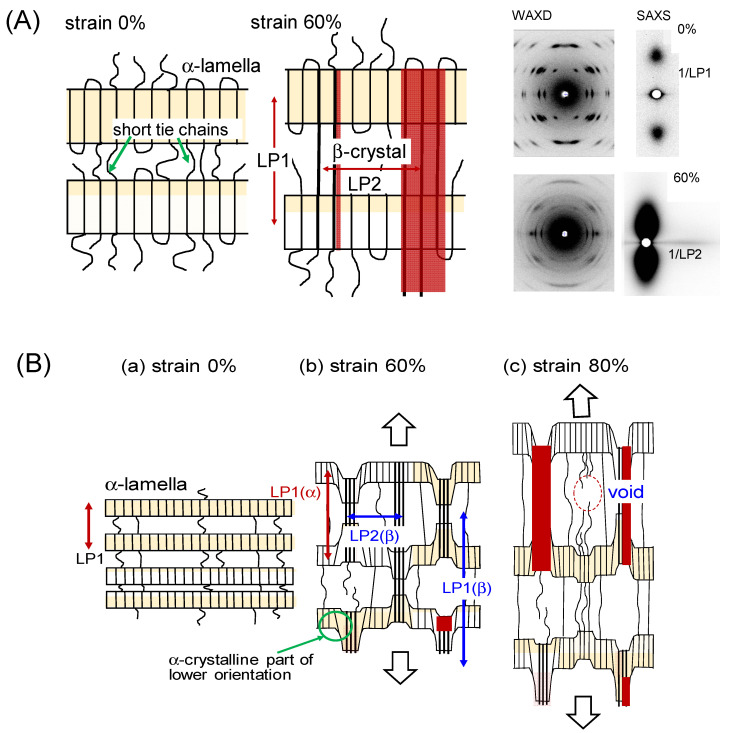
(**A**) Illustration of the higher-order structure of the α and β forms of the oriented PHB sample. (**B**) The higher-order structural change in the tensile deformation process of the oriented PHB sample. (**a**) The stacked lamellar structure of the initial α form with the short and long tie chains connecting the adjacent lamellae. (**b**) The tensile deformation induces the high extension of the short tie-chain segments, which transform to the β form. The lamellar domain of the α form connected to these strained tie chains is also transformed to the β form. The β-form bundles are arrayed side-by-side with the averaged long period LP2. (**c**) The further tension causes the breakage of the highly stressed short tie chains and generate the micro-voids, which grow to larger macro-voids and cause the breakage of the sample [23].

The β crystal modification of PHB is formed only under the application of a high tensile force. When the uniaxially oriented α form sample is tensioned at about 60% of the original length at room temperature, the mixture of these two crystalline phases is obtained (see Figure 7b) [23]. The perfect transformation to the β form is impossible, before which the sample is broken at last. It is impossible to obtain the 2D WAXD pattern of the pure β form. In order to obtain the diffraction pattern expected for the pure β form, the X-ray diffraction pattern of the pure α form was subtracted from the original diffraction pattern taken for the mixture sample. The result is shown in Figure 7c, from which the crystal structure model of the β form was proposed. The chains take the fully extended conformation and they are packed in the hexagonal unit cell with the space group *P*3_2_21. The tensile force causes the extension of the originally contracted 2_1_ helix of the α form. The streak lines detected on the X-ray diffraction pattern indicate the disorder of the relative height between the neighboring chains. The combination of the WAXD and SAXS (small-angle X-ray scattering) data revealed the transition mechanism viewed from the higher-order level. As shown in Figure 8, the short tie chains passing through the neighboring lamellae are extended by the strong tensile force and crystallize to the β form [23]. The molecular chains packed in the α-form lamellae are also forced to change to the extended β form. The remarkable change of the SAXS pattern, that is, the increase of the long period and the creation of many voids, can be interpreted by these structural changes. As the tensile force is increased furthermore, the extended chains are broken and the small voids increase in size. Finally, these voids are fused together to form the macro-voids, resulting in the fracture of the whole sample.

## 4. Crystal Structures of Synthetic Polymers by WAND Data Analyses

It has been limited to use the WAND data for the crystal structure analysis of synthetic polymers. This is because most of the structural discussion can be made by the X-ray diffraction data analysis. However, we cannot miss the weak point of X-ray diffraction method. As shown in Table 1, the X-ray scattering power (atomic scattering factor) becomes stronger in the order of total number of electrons contained in the atoms. It is almost impossible to determine the positions of H atoms in the crystal lattice, because H atom contains only one electron. From such a sense, we have a dilemma in the quantitative discussion of structure–property relation of polymers. As mentioned above, in order to interpret the anisotropic mechanical property, we have to clarify the H positions at high accuracy. The neutron diffraction data may play an important role in the dissolution of such a serious dilemma. As already mentioned, the D atom (not H atom) can scatter the coherent neutron signals with almost comparable power with C atom. In this way, one important motivation for the usage of WAND data is to determine the D (H) positions in the unit cell.

### 4.1. Orthorhombic Polyethylene

**Figure 9:**
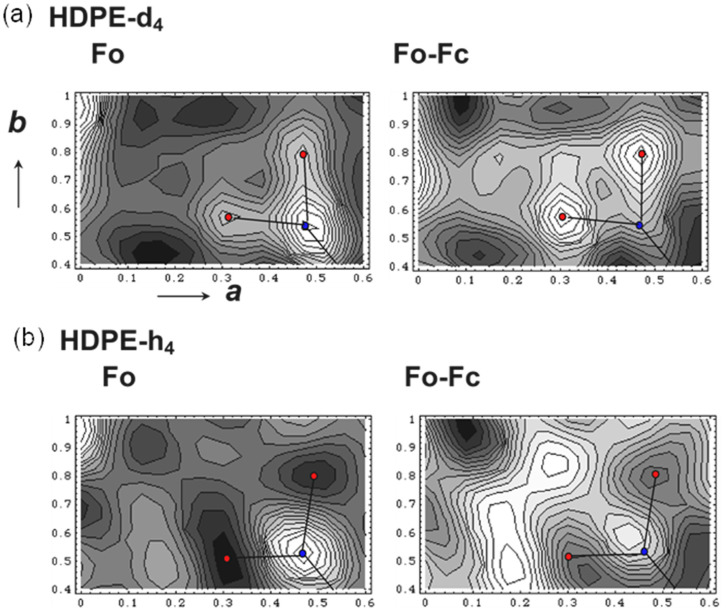
The two-dimensional maps of the nuclear density distributions in the unit cell of the orthorhombic polyethylene crystal. The difference Fourier (Fo–Fc) maps are also shown. (**a**) Deuterated PE (HDPE-d4) and (**b**) hydrogenous PE (HDPE-h4) (refer to Figure 4b,c) [24].

The 2D WAND patterns of the D- and H-orthorhombic PE samples are already shown in Figure 4. The Fourier-transform maps of nuclear density derived from the WAND data are shown in Figure 9. In HDPE-d4, the positive peaks of both of C and D atoms are detected clearly [24]. The difference Fourier transform obtained by subtracting the contributions of the C atoms gives the D atom peaks more clearly. On the other hand, the Fourier-transform map of the HDPE-h4 sample gives the positive C peaks and the negative H peaks (refer to Table 1). However, as shown in Figure 4, the observed diffraction peaks were not large in number to refine the structure.

A series of random copolymers of H and D-ethylene monomers were synthesized and their WAND patterns were measured. The systematic change of the diffraction pattern was reproduced quite well on the basis of the above-determined crystal structure. The details are referred to in the literature [25].

**Figure 10:**
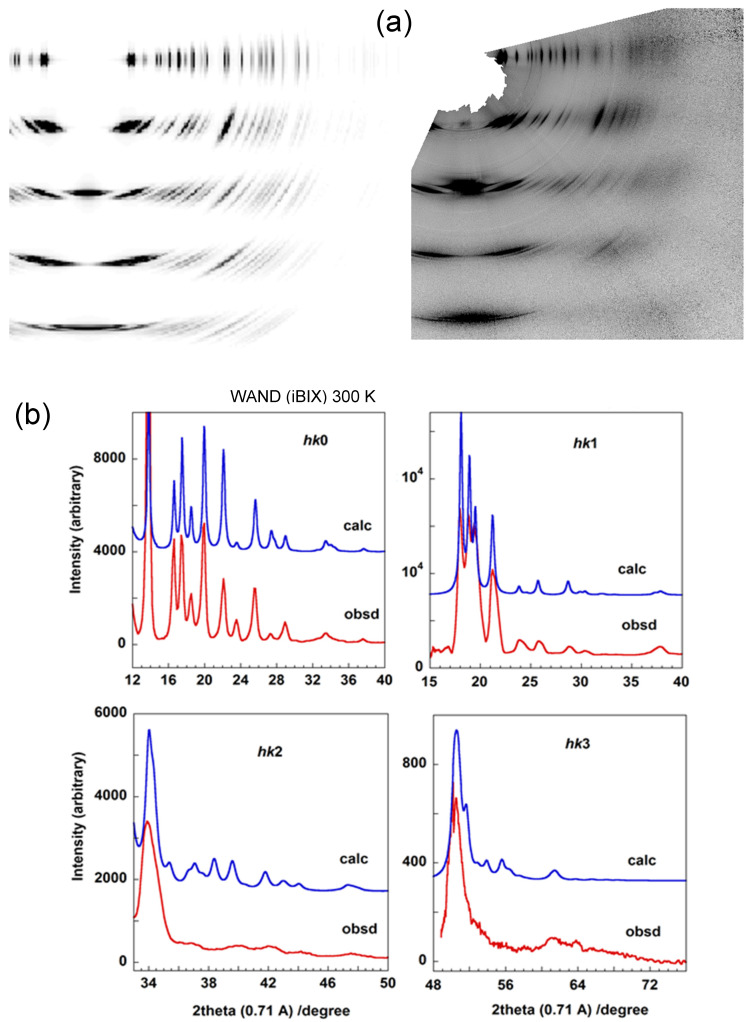
(**a**) 2D WAND pattern of HDPE-d4 sample measured at 300 K using iBIX system. The left pattern is the calculated one. (**b**) The comparison of the diffraction profiles along the various layer lines (L0–L3) between the observed and calculated ones.

The 2D WAND pattern of HDPE-d4 was measured also with the i-BIX system. As shown in Figure 10, the diffraction angle range was very wide, giving up to the 4th layer lines. The agreement between the observed and calculated diffraction profiles is nice for these layer lines.

### 4.2. Trigonal Polyoxymethylene

#### 4.2.1. Crystal Structure of POM

**Figure 11:**
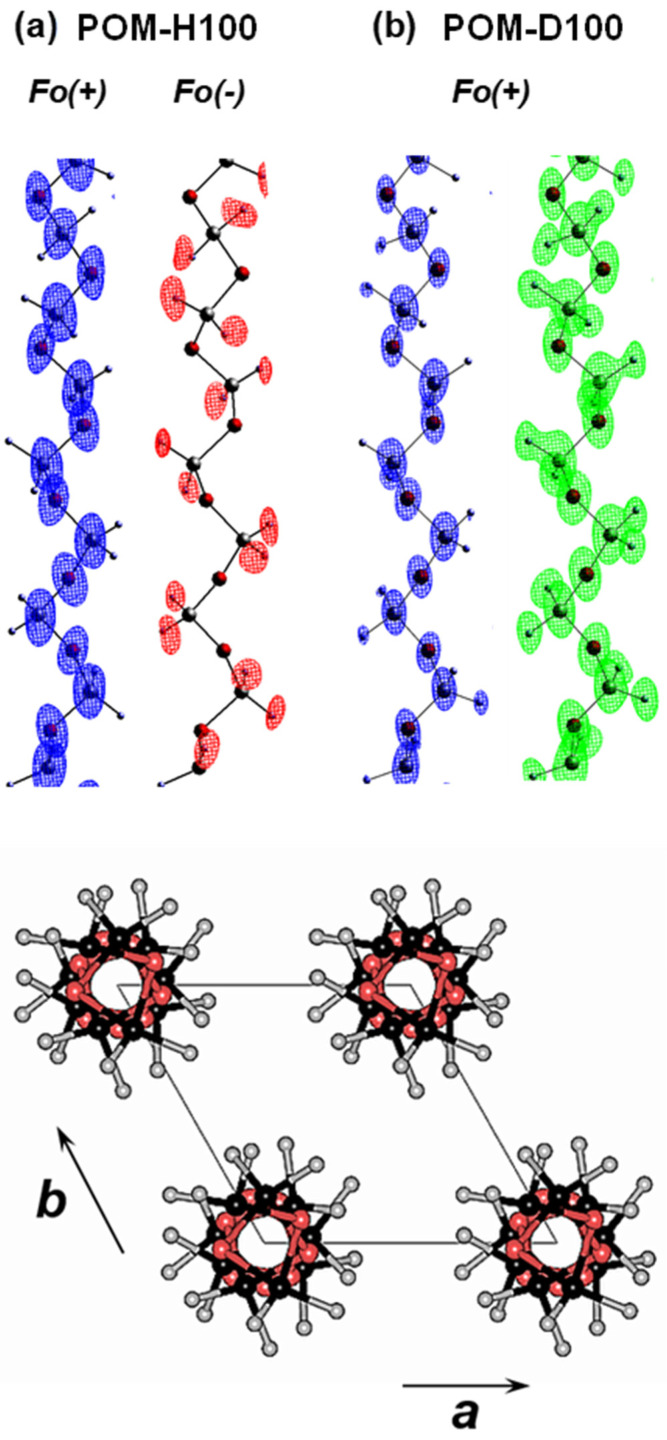
Chain conformation and crystal structure of POM. The nuclear density distributions revealed by the WAND data analyses are shown in (**a**) POM-H100 and (**b**) POM-D100, respectively. The positions of the light H atoms are detected as the negative peaks (*F*_o_(−)) in a wider diffraction angle range [27].

The hydrogenated polyoxymethylene (POM-H100, -[CH_2_O]_n_-) sample was obtained by the γ-ray-induced solid-state polymerization reaction of trioxane (or tetraoxane) needle-type single crystal. The thus-obtained POM-H100 sample is 100% crystalline and perfectly oriented; however, it is not a single crystal but a bundle of multi-crystals. The 2D WAXD pattern was measured by rotating the sample around the chain axis to erase the heterogeneous intensity distribution coming from such multiple crystals [26]. The data were collected using a high energy synchrotron X-ray beam and with the cylindrical imaging–plate camera as a 2D detector [27]. The quantitative analysis of the observed 500 diffraction peaks revealed the C, O and H atomic positions at high accuracy. The 2D-WAND data were obtained for both the POM-D100 (-[CD_2_O]_n_-) and POM-H100 crystals, where the deuterated POM samples were produced by the solid-state polymerization of deuterated-trioxane single crystals. The detailed analysis gave the D atomic positions, as shown in Figure 11. The thus-determined crystal structure allowed us to estimate the anisotropic 3D elastic constants matrix, from which the various mechanical properties were evaluated [13].

#### 4.2.2. D/H Random Copolymers

As one application, a series of blend samples of D-trioxane and H-trioxane monomers were prepared (H/D = 100/0, 70/30, 50/50, 30/70, and 0/100 molar ratio). The γ-ray irradiation was performed to synthesize the D/H copolymers. Are these polymers really random copolymers of the D and H monomeric units? The following experiment was performed.

**Figure 12:**
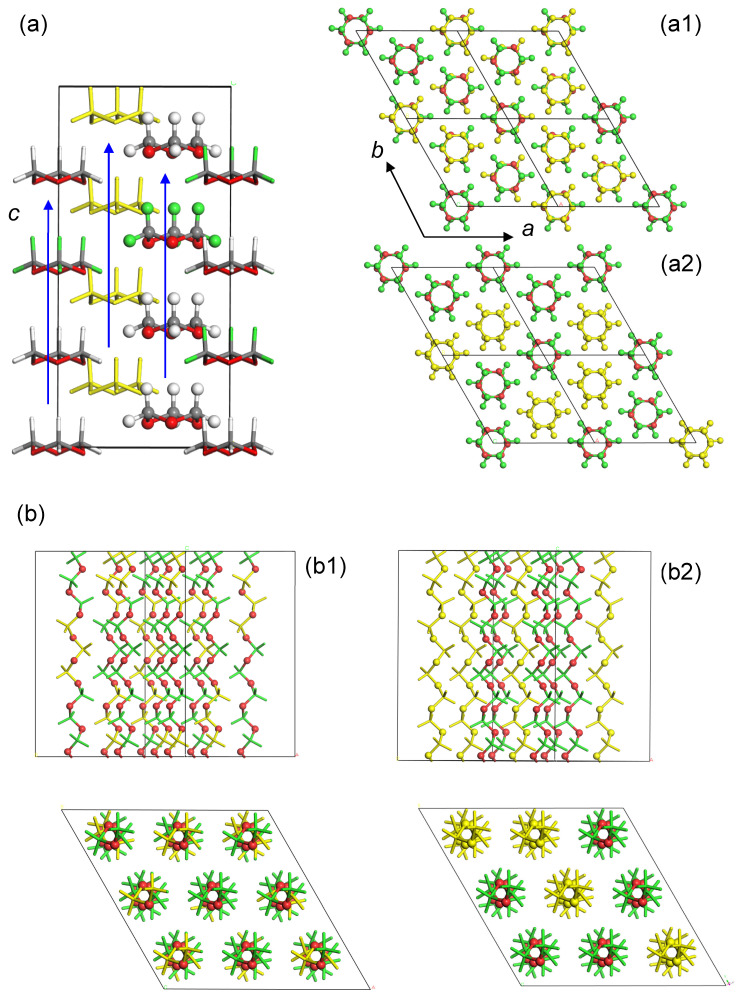
(**a**) Crystal structure of trioxane (TOX): (**a1**) random packing of D- and H-TOXs, (**a2**) random-packing model of D-TOX columns and H-TOX columns. The vertical blue arrows indicate the direction of the possible polymerization reaction. (**b**) The corresponding POM crystals: (**b1**) random arrangements of D/H monomeric units along the chain axis, (**b2**) random array of POM-D100 and POM-H100 chains.

The trioxane (TOX) monomers are volatile and they are put in a sealed glass tube. By leaving it at room temperature for a while, they crystalize into a cylindrical rod. The X-ray analysis revealed the crystal structure shown in Figure 12 [28]. The TOX is a polar crystal, where the polar ring molecules are packed with their dipoles in parallel to the *c* axis. The LO (longitudinal optical) and TO (transverse optical) vibrational modes were observed clearly in the Raman spectra [29]. The blend samples of the D- and H-TOX monomers form the similar single crystals. As illustrated in Figure 12a, these isotopic monomer species might be packed in the crystals in the several possible modes: (a-1) the D and H rings are packed randomly in the crystal lattice; (a-2) the D (or H) rings are stacked regularly along the *c* axis to form a columnar rod, and these D (or H) rods are packed randomly in the crystal lattice, and (a-3, not shown) the D- and H-monomers are perfectly separated to form the individual domains.

**Figure 13:**
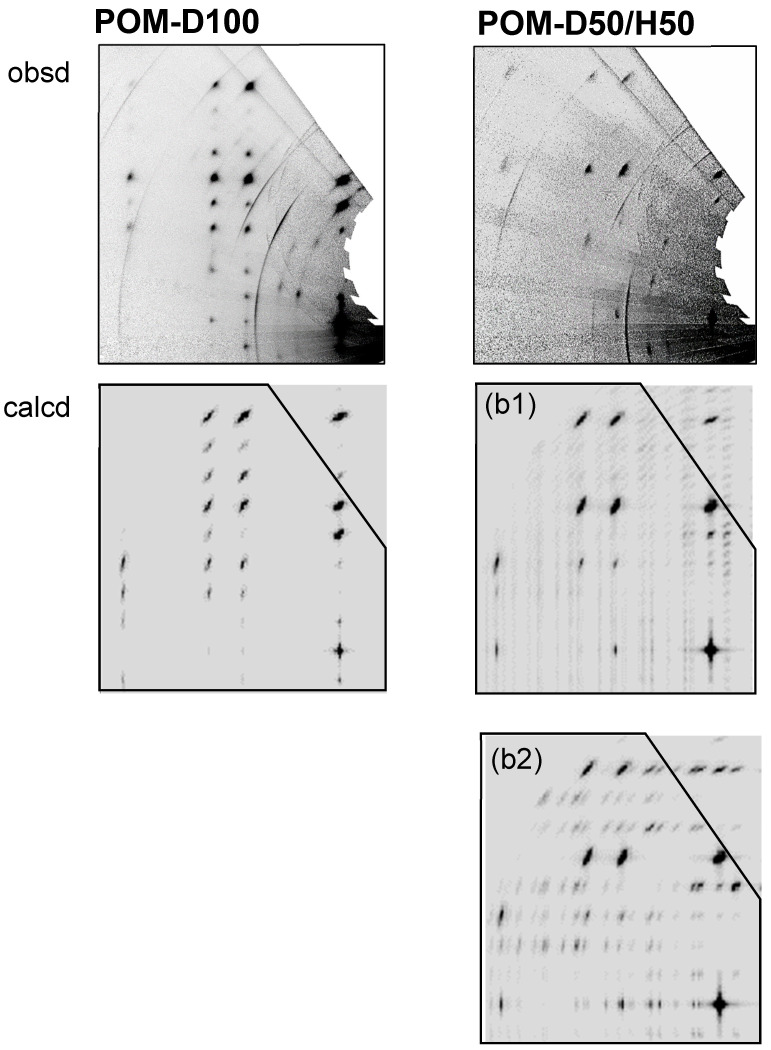
Comparison between the observed and calculated 2D WAND patterns of POM-D100 and POM-D50/H50 copolymer samples. The (**b1**) and (**b2**) correspond to the models shown in Figure 12.

The γ-ray polymerization reaction of the D/H-TOX blend samples gave the crystalline polymer samples containing the D and H monomeric units. The polymerization reaction of TOX occurs along the *c* axis, as indicated by the blue arrows in Figure 12a. In the case of the D/H-blended TOX crystal with the regular packing structure shown in Figure 12(a2), the produced polymer sample may consist of the mixture of POM-D100 and POM-H100 chains as illustrated in Figure 12(b2). The IR spectra might be a simple overlap of the two spectra intrinsic to these different types of chains. The band positions in the actually observed IR data were shifted depending on the D/H content because of the vibrational couplings (not shown here), inconsistent with the above-mentioned prediction. Then, we might have the random copolymers as shown in the model Figure 12(b1). The spatial distribution of the D and H monomeric units can be checked by the WAND data. Among the various models, the random copolymer model was found to reproduce the observed fiber pattern well, as shown in Figure 13. In the observed WAND patterns, the relative intensity of the diffraction peaks changes differently between the D100 and D50/H50 samples. The WAND pattern of the random copolymer model (D50/H50), which was calculated using a commercial software Cerius^2^ (Biovia), was similar to the actually observed pattern of the D50/H50 POM sample. The mixture model of POM-D100 and POM-H100 chains in the common crystal lattice Figure 12(b2) cannot reproduce the observed data well. The conclusion is as follows: the D- and H-trioxane molecules are packed randomly in the crystal lattice, and the γ-ray-polymerized POM crystal consists of the parallel array of the D/H random copolymer chains. (It must be noted that the trioxane molecule has a triple sequence of CH_2_O units and so the random copolymers must be treated as the random arrays of (CH_2_O)_3_ and (CD_2_O)_3_ segments in the model building process.)

**Figure 14:**
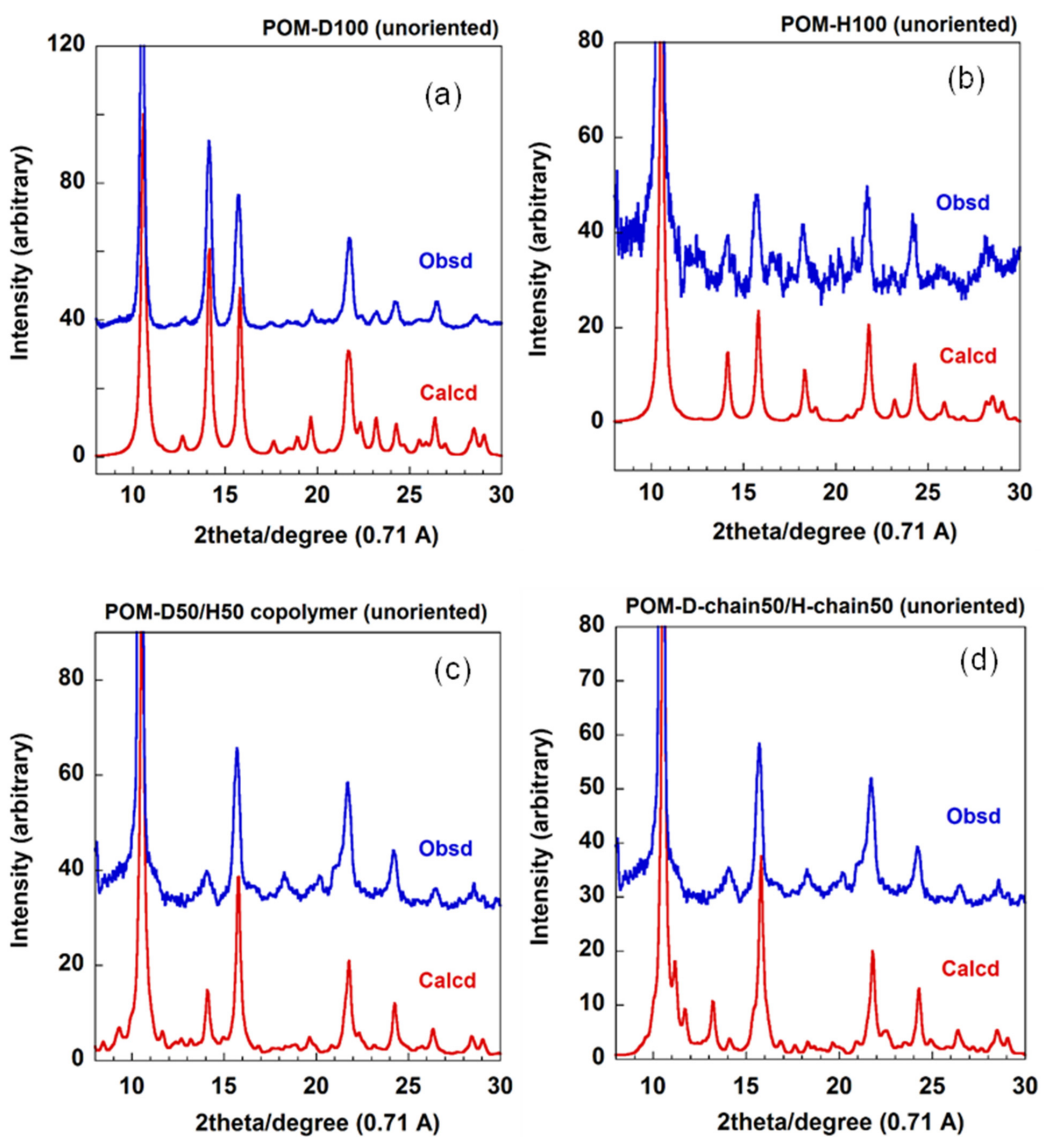
(**a**) WAND profiles of unoriented POM samples: (**a**) deuterated POM-D100, (**b**) POM-H100, and (**c**) POM-D50/H50 random copolymer. These observed data are compared with the profiles calculated for POM-D100, POM-H100, and POM-D50/H50 random copolymer (Figure 12(b1)), respectively. (**d**) The hypothetic model consisting of POM-D100 chains and POM-H100 chains in the lattice at 50% ratio, which is compared with the 50D/50H copolymer sample (refer to Figure 12(b2)).

In order to confirm this conclusion, the WAND powder patterns were measured in a wider diffraction angle range for a series of D/H random copolymers, which were synthesized in the homogeneous solutions [30] and slowly cooled from the molten state to obtain the unoriented samples. The thus-measured diffraction profiles were compared with those predicted for the various models. The pure POM-D100 and POM-H100 gave a good agreement between the observed and calculated profiles (Figure 14a and Figure 14b, respectively). The random copolymer model of the D- and H-monomeric units along the chain axis (Figure 12(b1)) reproduced the WAND profile observed for the D50/H50 copolymer sample nicely [Figure 14c].

## 5. Dilemma between the WAXD and WAND Analyses

The above-mentioned several examples of the crystal structure analyses of synthetic polymers suggest that they confirm the consistency between the WAXD and WAND methods. It might be indisputable to say that the X-ray structure analysis gives the reliable crystal structures of synthetic polymers as long as the total number of the observed diffractions is relatively high. In other words, we have a plausible reason why people have performed mainly the X-ray data analyses to extract the crystal structure information of synthetic polymers. The neutron facility is one of the so-called big sciences, and much care is needed for the collection of the WAND data. Moreover, the WAND measurements still have many unsolved problems: the deuterated polymers must be synthesized; the large samples of a few mm^3^ size must be prepared; the more sensitive detectors are required for the data collection of higher S/N ratio; the power of the neutron source is needed to increase furthermore. Judging from these realistic situations, the WAND data analysis may be assumed as the secondary (or comprehensive) tool as far as the crystal structure analysis of synthetic polymers is concerned.

However, this passive opinion is not necessarily true for a kind of polymer species. Recently, we have encountered a serious problem, in which the structure model derived by the X-ray analysis at a high accuracy could not reproduce the observed WAND data at all. This is the case of the structure analysis of at-PVA and its iodine complex [31].

### 5.1. Crystal Structure of at-PVA

**Figure 15:**
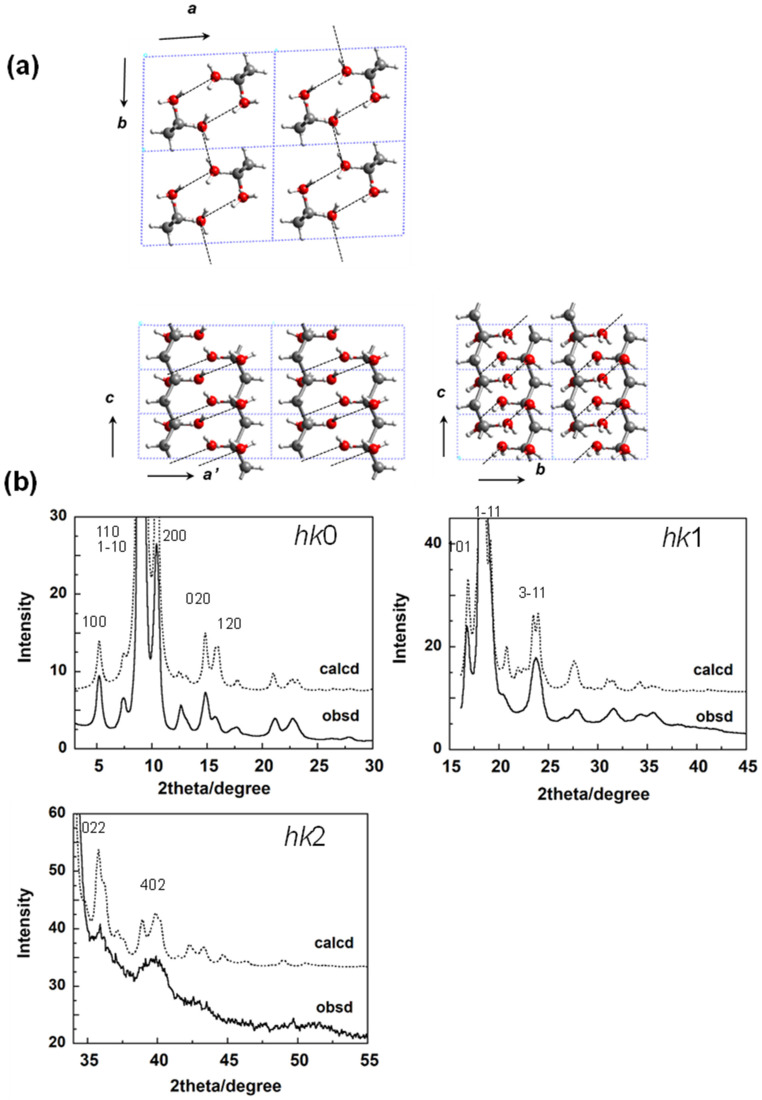
(**a**) Crystal structure of at-PVA derived by the WAXD data analysis, and (**b**) the comparison of the observed X-ray data with those calculated for the structure model (**a**). The Mo-Kα line with 0.71 Å wavelength was used as the incident X-ray beam [31].

The history of crystal structure analysis of *atactic* poly(vinyl alcohol) (at-PVA, [-CH_2_HC(OH)]_n_-) is quite long. Since polymer science started about a century ago, the crystal structure of at-PVA was reported in many papers using the X-ray analysis. One model is shown in Figure 15a, which was proposed by Bunn [32] and supported by the Japanese group [33]. The polarized Raman spectra measured for the doubly oriented sample were consistent with this structure model [34]. However, another structure model was proposed also with the X-ray diffraction data analysis by the other Japanese group [35]. In the former model, the planar-zigzag chains are packed in the unit cell so that the intermolecular hydrogen bonds are formed between the neighboring chains along the 110 plane and along the *b* axis, as shown in Figure 15a. In the latter model, on the other hand, the zigzag chains are arrayed along the *a* axial direction with the intermolecular hydrogen bonds. In many works, the former model has been used as the crystal structure of at-PVA. However, the latter model is still quoted in some papers. We needed to establish the structure. We measured the WAXD data again using a Mo-Kα X-ray beam of shorter wavelength, which was helpful to collect more diffraction data [31]. The observed diffraction data, in particular, the layer line profiles were not reproduced very well by the latter model. Therefore, the former crystal structure model (Figure 15a) may be usable for at-PVA as long as the WAXD data covering the *hkl* diffractions of *l* = 0~2 are analyzed. Before the WAND data analysis of at-PVA is mentioned, it might be better to learn about the structure of at-PVA-iodine complex.

### 5.2. Crystal Structure of at-PVA-Iodine Complex

**Figure 16:**
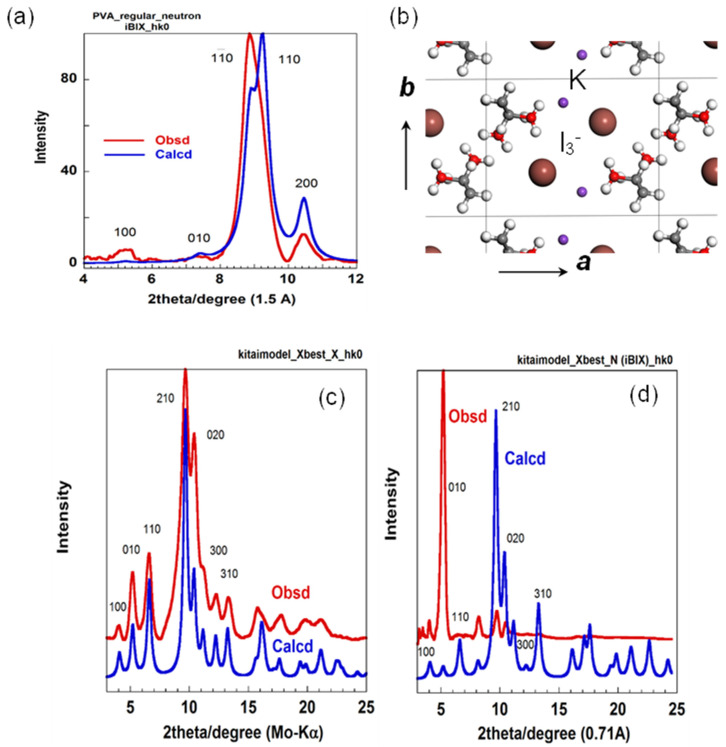
(**a**) Comparison of the observed and calculated WAND equatorial line profile for deuterated at-PVA sample, where the calculation was performed for the crystal structure model derived by the WAXD data analysis (refer to Figure 15). (**b**) Crystal structure model of at-PVA-iodine complex (form II) derived from the WAXD data analysis [37]. (**c**) Comparison of the observed WAXD equatorial line profile of at-PVA-iodine complex (II) with that calculated for the X-ray analyzed structure model (**b**). (**d**) Comparison of the observed WAND equatorial line profile of at-PVA-iodine complex (II) with that calculated using the X-ray derived model (**b**) [31].

When the oriented at-PVA sample is immersed into a KI/I_2_ aqueous solution, the so-called at-PVA-iodine complex is formed in the amorphous region and also in the crystalline phase, the formation behavior of which is dependent on the iodine concentration [36]. Here, we focus on the case of the highly concentrated solution (3 mol/L). The thus-prepared complex is named form II [37,38]. As shown in Figure 16c, the X-ray diffraction pattern from form II gives many sharp peaks along the equatorial line. In the 2D pattern (not shown here), the diffuse streaks are observed on the layer lines, which originate from the disorder of the relative height of the neighboring I_3_^-^ ion columns [37]. The equatorial line profile allows us to deduce the structure of the complex projected along the chain axis. The thus-determined structure model is shown in Figure 16b, which gave the excellent agreement between the observed and calculated X-ray diffraction profiles (Figure 16c). The quantum mechanical calculation performed using this structure model revealed the formation mechanism of the complex (that is, the charge-transfer mechanism) [39].

### 5.3. The Combination of WAXD and WAND Data Analysis

In this way, the crystal structures of at-PVA and its iodine complex (form II) were determined by the quantitative analysis of the WAXD data. As you notice, however, the thus-determined structures may always have some ambiguity because the observed diffraction data were quite limited in number. Then, we tried to check these structures on the basis of the WAND data analyses. The WAND patterns were measured using the deuterated at-PVA sample and its iodine complex (form II) [31]. The WAND data were measured using the above-mentioned two types of the neutron diffractometers so that any inconsistency was not remained in the neutron data themselves. The thus-obtained WAND data were compared with the profiles predicted for the crystal structure models shown in Figure 15a and Figure 16b. For the at-PVA sample, the agreement between the observed and calculated data was not very bad. However, one discrepancy was detected in the relative intensity of the most intense equatorial-line peaks (110 and 1$\bar{1}$0) (Figure 16a). Once we focused on the WAND equatorial-line profile of the at-PVA-iodine complex, as shown in Figure 16d, we were shocked to find a great difference of the observed data from the profile predicted for the above-mentioned iodine-complex structure model in spite of the fact that this model gave a good agreement with the observed X-ray diffraction profile!

**Figure 17:**
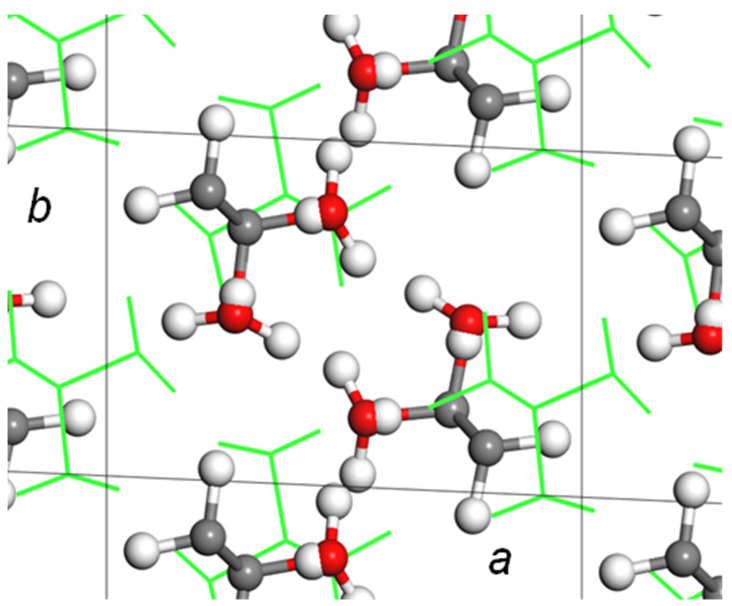
Introduction of packing disorder to the crystal structure of at-PVA shown in Figure 15a. The original unit cell was shifted by 1/2 along the 110 diagonal plane [31].

**Figure 18:**
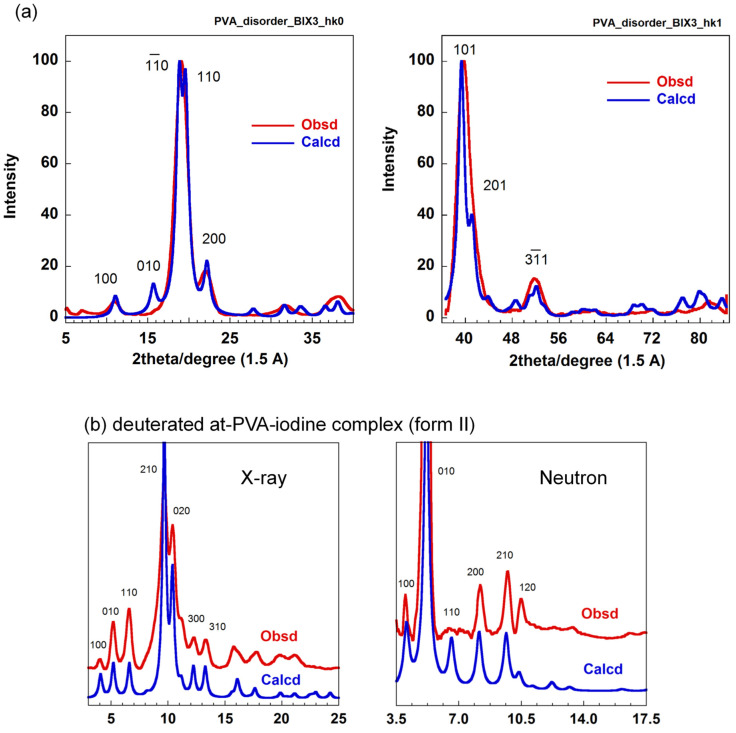
(**a**) Comparison of the observed and calculated WAND line profiles (equatorial and first-layer lines) for deuterated at-PVA sample, where the calculation was performed for the statistically disordered crystal structure model (refer to Figure 17). (**b**) Comparison of the observed WAND equatorial line profile of at-PVA-iodine complex (II) with that calculated for the statistically disordered model shown in Figure 19 [31].

After many trial-and-error processes, we found one model to satisfy both of the observed WAXD and WAND data consistently; an introduction of a packing disorder. In the case of at-PVA itself, the new structure model assumes the statistical disorder of the two unit cells by shifting d_110_/2 along the 110 plane (the two structures of gray and green colors in Figure 17). This disorder model may be imagined as the statistical aggregation of the translationally shifted domains, where the local structure is the same among the domains. These domains are coherent with each other and the diffraction profile is affected by modifying the aggregation mode of the domains. As shown in Figure 18a, the relative intensity of the 110 and 1$\bar{1}$0 peaks detected in the WAND profiles of the at-PVA itself has been revised better, compared with that of the regular model (Figure 16a). This type of domain-aggregation disorder introduced into the at-PVA crystalline region should be remained even when the iodine complex is formed after immersing the at-PVA sample into the iodine solution. In fact, as shown in Figure 18b, the equatorial line profile observed for the at-PVA-iodine complex form II has been also reproduced quite well for both the WAXD and WAND data [31].

The origin of this dramatic story comes from the difference in the atomic scattering power between the X-ray and neutron beams. The at-PVA-iodine complex is made of the atomic species of C, O, H, K, and I elements. As shown in Table 1, the X-ray scattering power of an atom is proportional to the number of electrons, so the iodine ions give an overwhelmingly large contribution to the observed X-ray diffraction intensity (I >> K > O~C >> H). Therefore, the information extracted from the WAXD data analysis is mainly about the positions of iodine atoms in the unit cell. On the other hand, the neutron scattering power is not simply dependent on the atomic number, but it changes in a complicated way; the so-called neutron scattering cross sectional area is C~O~D >> K and I. The neuron data analysis can give the positional information about the at-PVA chains mainly. Therefore, even if the position and orientation of the at-PVA chains is wrong in the structural model of the iodine complex, it is difficult to find these errors by the X-ray structure analysis only. Once the X-ray-derived model is adopted in the WAND data analysis, we notice for the first time the inconsistency of the X-ray-derived model, as found in Figure 16. In the case of at-PVA and its iodine complex, the introduction of the statistically disordered structure has been able to solve such a dilemma between the WAXD and WAND data analyses, as mentioned above.

**Figure 19:**
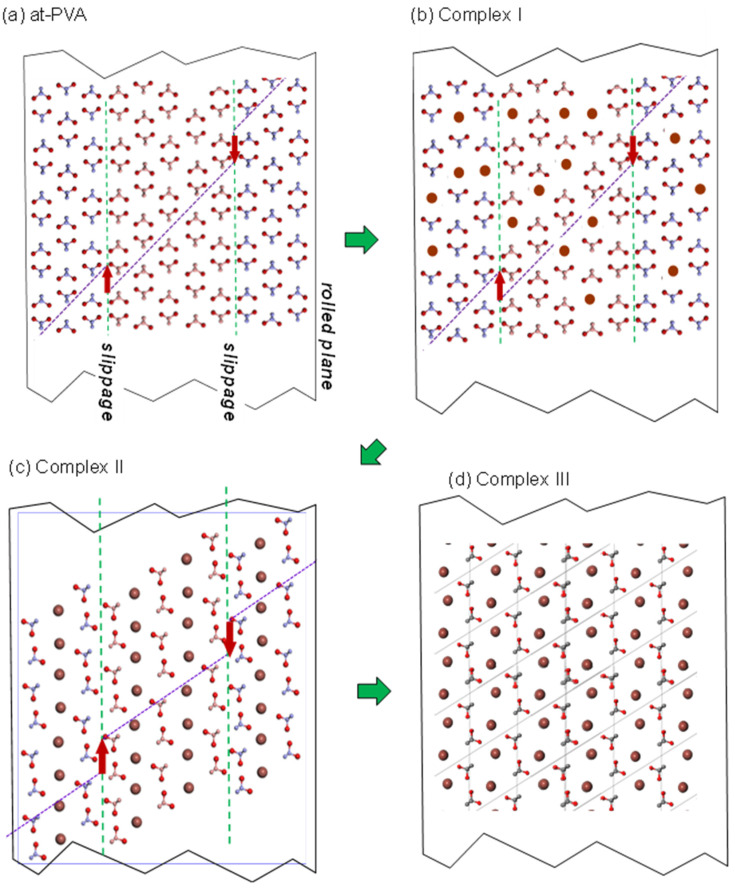
Schematic structural transformation of at-PVA to a series of at-PVA-iodine complexes (forms I, II, and III) which are prepared by immersing the pristine at-PVA sample into the iodine solutions of the different concentrations) [38]. (**a**) The statistically-randomly-slipped domain structure of at-PVA crystal. (**b**) It changes to the iodine complex I by immersing in the 0.1~0.8 M KI/I_2_ solution, (**c**) to the iodine complex II in the 1~3 M KI/I_2_ solution, and (**d**) to the iodine complex III in the 3 M HI/I_2_ solution.

**Figure 20:**
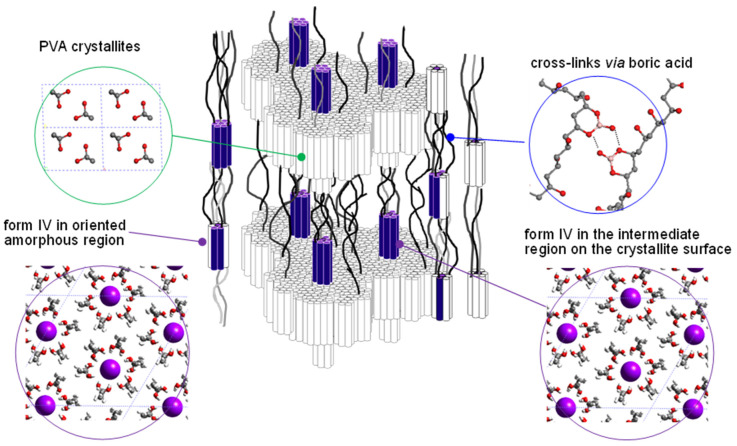
Structural model of a polarizer. The crystalline lamellae are stacked along the drawn direction. The complex form IV is created in some of the oriented amorphous regions. The oriented amorphous chains are existent near the top and bottom surfaces of the crystallites and also as the extended tie chain segments (or the extended rigid amorphous chains) in the normal amorphous regions. Industrially, the at-PVA sample is also immersed in the boric acid solution. The cross linkages are formed in some parts of the amorphous regions through the chemical bonds between the boric acid molecules and the hydroxyl groups of at-PVA chains, which stabilize the complex structure [38].

As summarized in Figure 19, four different types of the at-PVA-iodine complex have been found out so far; forms I, II, III, and IV [38]. The form IV is produced in the oriented amorphous region immersed into a dilute iodine solution, which is the structural origin of the optical polarizer. The I_5_^-^ ions are surrounded by the 6 at-PVA chains, and these groups are packed in the hexagonal mode (see Figure 20). The other three forms are formed in the crystalline regions. The structural transformation occurring in the at-PVA sample immersed in the iodine solution is illustrated in Figure 19 with the above-mentioned disordered aggregation of the domains taken into account, where the packing mode of iodine ions and the relative orientation of at-PVA chains are changed stepwise depending on the immersion time and/or the iodine concentration.

## 6. Distribution of the Bonded Electron Density along the Polymer Chain (X-N Method)

As mentioned in the introductory section, we can obtain the so-called giant single crystal of polymer in a lucky case. For example, a large single crystal of diacetylene compound is prepared by the solid-state photo-induced polymerization reaction of the corresponding monomer single crystal [40]. The thus-prepared polymer single crystal gives the several thousands of sharp diffraction spots, from which the details of the atomic arrangement in the crystal lattice can be derived at high accuracy. In a case, we may know the atomistic mechanical deformation mechanism of polymer chain subjected to a tensile force [41]. In a luckier case, we can extract the information of the electron density distribution by combining the WAXD and WAND data [42].

**Figure 21:**
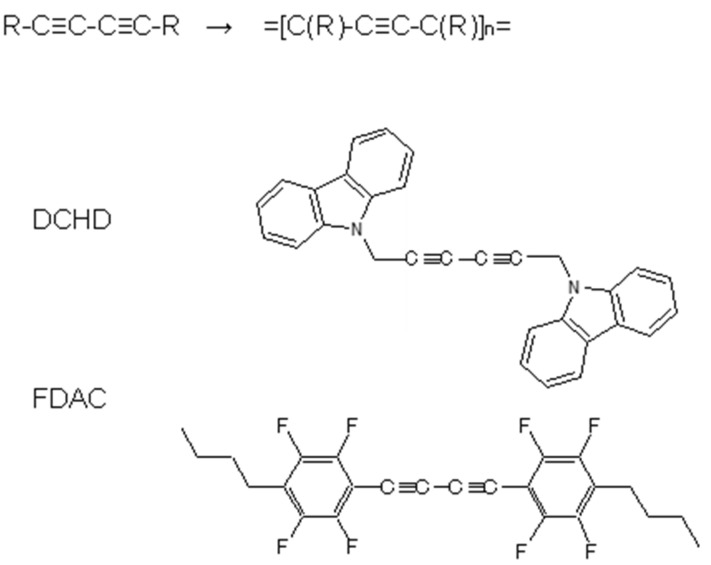
Chemical formulae of diacetylene monomers. DCHD: 1,6-di(N-carbazolyl)-2,4- hexadiyne. FDAC:1,4-di(4-butyl 2,3,5,6-tetrafluorophenyl)diyne. The irradiation of light induces the polymerization reaction in the solid state.

The polymer single crystals focused on here are polydiacetylene compounds. Depending on the side groups, the degree of electronic conjugation between diacetylene and side groups is more or less different. The diacetylene compounds treated here are shown in Figure 21. The DCHD molecule has CH_2_ units between the diacetylene and carbazolyl group, and so the electronic conjugation between the skeletal chain and the side groups is cut off. In the FDAC case, some degree of conjugation is expected to occur between them.

### 6.1. Photoinduced Solid-State Polymerization of FDAC

**Figure 22:**
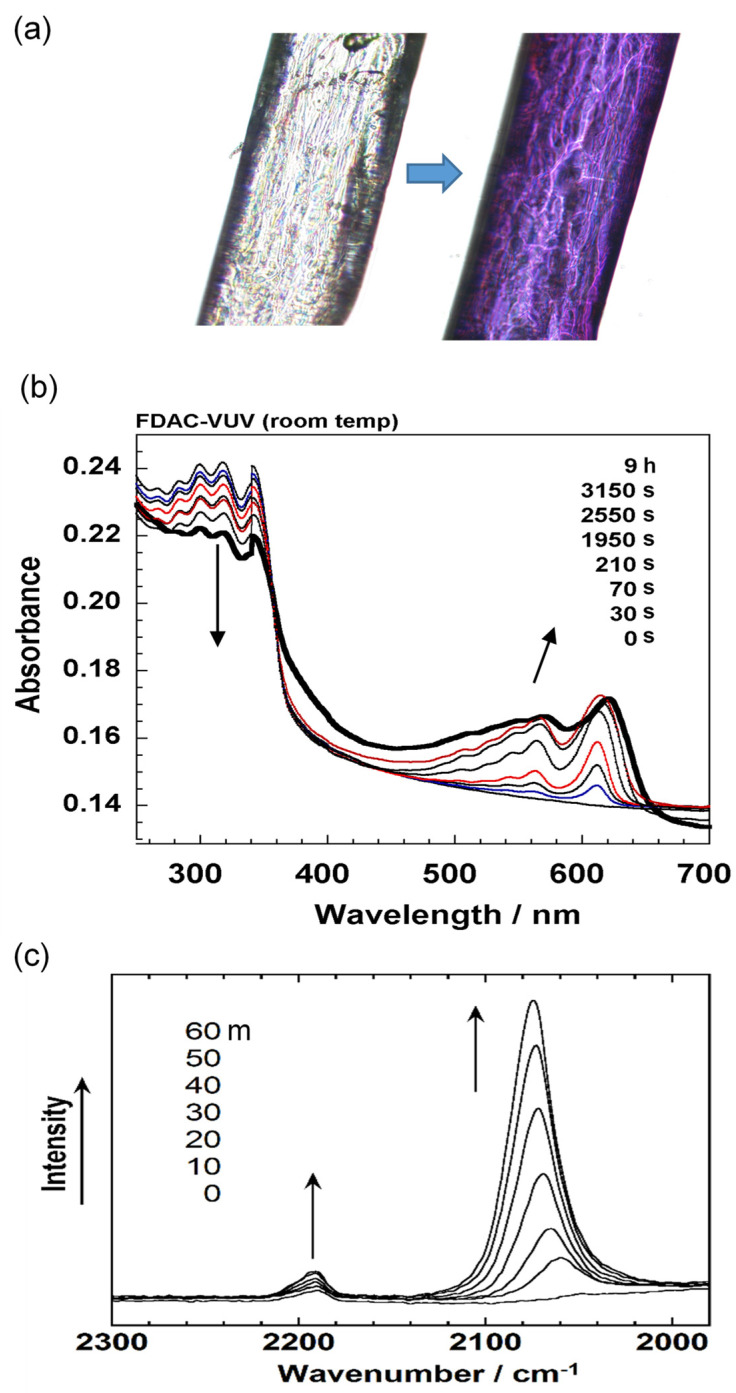
(**a**) A single crystal of FDAC before (non-color) and after (purple blue) the irradiation of a fluorescence light at room temperature. (**b**) UV–irradiation-time dependence of the UV–Vis absorption spectra of FDAC. The bands at around 300 nm decreased in intensity, while the bands in the 500–600 nm region increased in parallel. (**c**) Time dependence of the resonance Raman spectra of FDAC, where the incident laser beam (532 nm wavelength) played as the irradiation light of the reaction and as the excitation beam of Raman spectral measurement [44].

**Figure 23:**
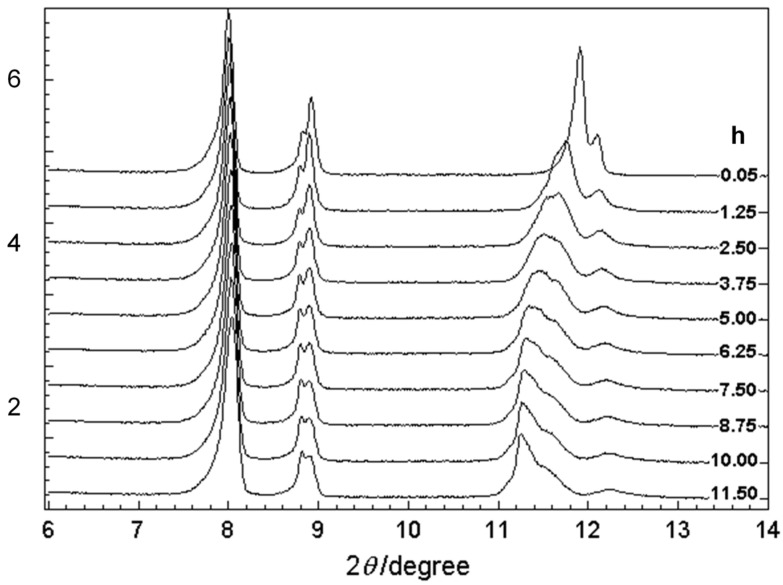
Change of the X-ray diffraction pattern of the FDAC powder sample in the progress of the polymerization reaction, where the X-ray beam (Cu-Kα, 1.54 Å) worked as the reaction stimulator and as the incident beam for the diffraction profile measurement [44].

**Figure 24:**
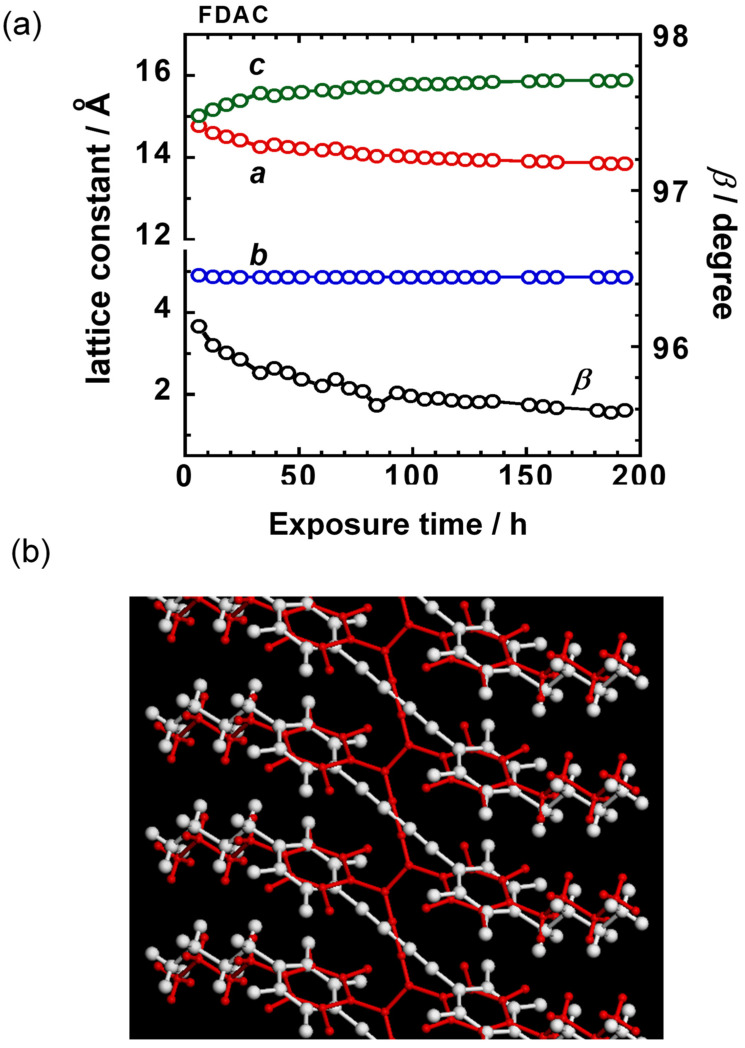
(**a**) The time dependence of the unit cell parameters obtained by the WAXD data analysis of FDAC single crystal under the X-ray irradiation process. (**b**) The crystal structures of monomer (white) and polymer (red).

**Figure 25:**
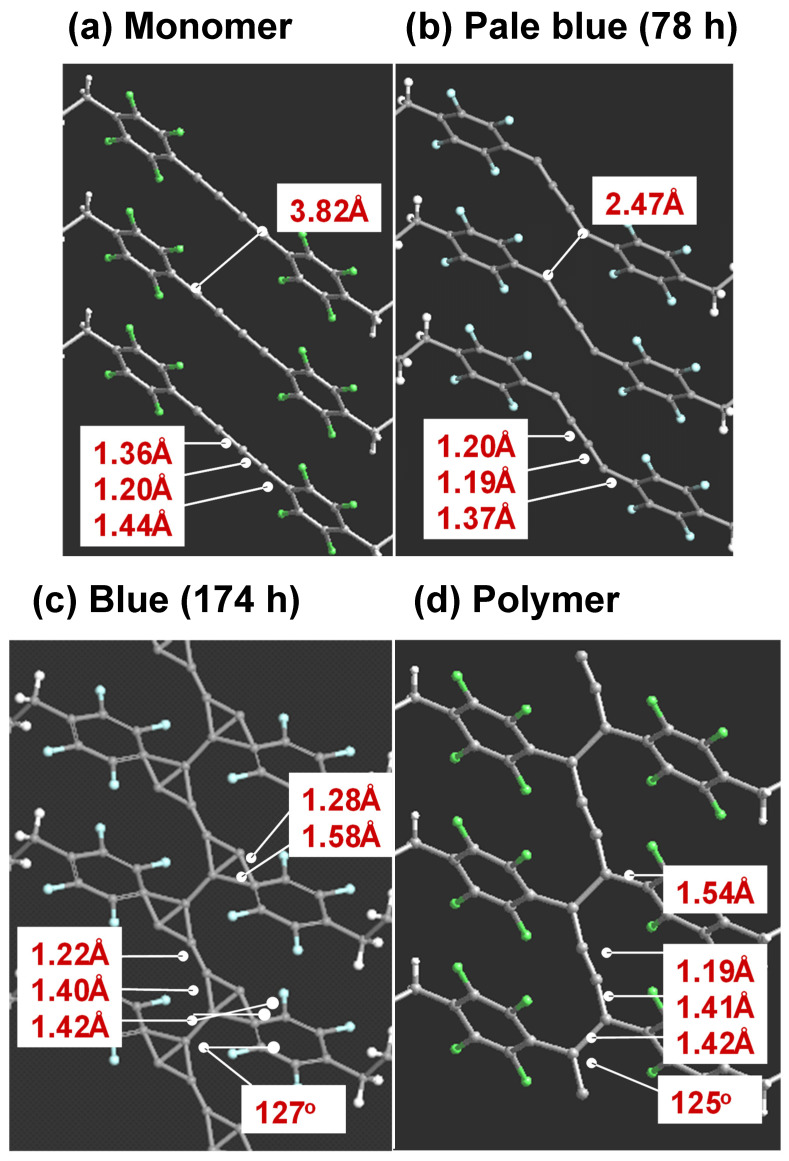
The crystal structure change of FDAC under the X-ray irradiation process. The models shown here are the averaged structures derived by the analyses of all the X-ray diffraction peaks.

**Figure 26:**
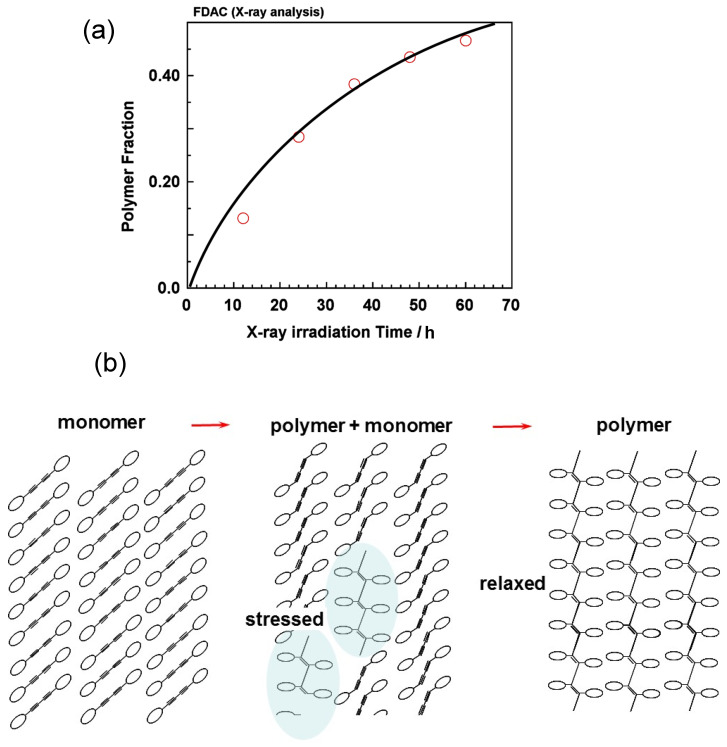
(**a**) Irradiation time dependence of the relative content of poly(FDAC) evaluated by the X-ray data analysis. (**b**) The illustration of the structural changes occurring in the crystal lattice of FDAC monomers.

Before the detailed structure analysis is described, it might be better to figure out the polymerization reaction itself under the photon irradiation. As an example, we will see here the case of FDAC [43]. The single crystal of FDAC was prepared from the petroleum ether solution in the dark room. The crystal was transparent and non-colored. When the crystal was exposed to a fluorescent lamp, the crystal changed to a purple blue color as shown in Figure 22a, where the crystal shape was almost kept unchanged even after a long time of irradiation. The UV–visible spectra changed remarkably during this process (Figure 22b). The original monomer crystal showed the absorption peaks in the UV region (250–350 nm) and no absorption was detected in the visible region (450–650 nm). The reacted crystal absorbed the visible light in the green-to-red color region, corresponding to the observed purple blue color of the crystal. The IR and Raman spectra were also observed to change remarkably. In particular, in the Raman spectra, the so-called resonance Raman phenomena occurred since the green laser of 532 nm wavelength was used, which was included in the UV–Vis absorption region. Once the sample was irradiated, the strong C≡C stretching band started to be detected at around 2060 cm^−1^, which is assigned to the reaction products. As the irradiation time was increased, the peak height increased and its position shifted gradually toward the higher wavenumber side, about 2080 cm^−1^. The remarkable change was also observed in the X-ray diffraction measurement (see Figure 23). In this way, the structural change was detected to occur with the remarkable change of the various data. In order to know the details of the structure change, the X-ray structure analysis was performed by collecting the diffraction data at the constant time intervals during the long reaction process, where the incident X-ray beam (Mo-Kα, 0.71 Å) worked as the monitor of the structure changes and as the excitation photon of the reaction (a similar experiment was performed also using the synchrotron X-ray beam). At the initial stage of reaction, the monomer molecules are packed in the monoclinic unit cell of *a* = 14.78 Å, *b* = 4.91 Å, *c* = 15.02 Å, and *β* = 96.13°. The crystal structure is shown in Figure 24b. On the way of reaction, the cell parameters changed apparently continuously as shown in Figure 24a. At the final stage, the unit cell changed to that of the parameters *a* = 13.83 Å, *b* (chain axis) = 4.85 Å, *c* = 15.80 Å, and *β* = 95.63°. The monomeric units are related by the space group symmetry of *P*12_1_/*n*1. From the comparison of the molecular packing mode between the initial and final structures, we can say clearly that the polymerization reaction occurs through the formation of covalent bonds between the neighboring monomer molecules (Figure 24b). The irradiation time dependence of the crystal structure was investigated by performing the analyses of a series of X-ray diffraction data collected in the various time regions. Figure 25 shows the changes of the averaged structure containing both the monomer and polymer components. After the X-ray irradiation for 78 h, the slight deformation of the monomer molecules occurred (Figure 25b). At about 174 h from the start, the two possible bonds were extracted, which correspond, respectively, to the inner bonds of the original monomer molecules and the covalent bonds connecting the neighboring monomer units (Figure 25c). The final structure is the polymer chain molecules (Figure 24b and Figure 25d). In an approximate way, the diffraction data might be simplified as the mixture of those of monomer and polymer species. This approximation gave us the change of the polymer fraction with the irradiation time, as shown in Figure 26a. However, this type of analysis seems to ignore the sensitive structural changes, and it does not give us the detailed information about the geometrical change of the monomeric unit itself.

Apart from the X-ray data, one of the good ideas to trace the reaction process is to observe the Raman spectral change as a function of time. As mentioned above, once the polymerization reaction started, the C≡C stretching band appeared and increased the intensity. However, we have to notice that the peak position actually shifted toward the *higher* frequency side (Figure 22c). The similar observation was reported for the other kind of diacetylene [44] and for *cis-cis*-diethyl muconate [45]. In general, the vibrational modes of the skeletal chains are shifted toward the lower-frequency side when the chains are tensioned strongly along the chain axis [46]. The above-detected higher-frequency shift of the C≡C band is totally opposite and interpreted in the following way. The concrete images are illustrated in Figure 26b. At first, we have to notice that the repeating period along the *b* axis is 4.91 Å for the monomer crystal, which is longer than the period of the final polymer product, 4.85 Å. The short polymer chain segments produced in the early stage are highly tensioned, because they are surrounded by many monomer molecules with the longer repeating period. The tensile stress working on the polymer chain segments are gradually relaxed with the progress of reaction. The long polymer chains produced at the final stage are totally free of the tensile stress. In this way, the gradual higher-frequency-shift of the Raman C≡C peak can monitor the relaxation of a tensile stress on the produced polymer chain segments.

### 6.2. Bonded Electron Density Distribution along the Polymer Chain

The above-mentioned large single crystals of polydiacetylene may allow us to perform the detailed study about the electron density distribution. The X-ray structure analysis revealed the fully extended conformation of polydiacetylene chains in the crystal lattice. Therefore, the electronic conjugation along the chain axis is predicted to develop to a high degree. This electron conjugation can be estimated quantitatively by the coupling of WAXD and WAND analyses or by using the so-called X-N method [47].

The wide-angle neutron diffraction data analysis reveals the positions of the atomic nuclei, while the X-ray diffraction data give the information about the center positons of electron density distributions, since the X-ray waves are scattered by the electron clouds. Strictly speaking, the positions of the centers of mass are different between the atomic nuclei and the electron clouds. If we imagine the formation process of a molecule, the atoms having the spherical electron distributions approach each other and create the covalent bonds by exchanging the electrons with each other. The electron clouds are shared by the two neighboring atoms and the distributions are deformed more or less from the original spherical shapes. In other words, the change of electron density distribution after the covalent bond formation may be evaluated from the slight difference of the positons between the atomic nuclei (deduced by the WAND analysis) and the electron density distributions (by the WAXD analysis). The concrete process is made in the following way.

(i) The whole electron density distribution ($\rho {\left(x\right)}^{X-ray}$) is calculated by the Fourier transform of the structure factors ($F_{obs}{\left(q\right)}^{X-ray}$) obtained by the X-ray analysis,

(4) $$\rho {\left(x\right)}^{X-ray}=\left(1/V\right)\int F_{obs}{\left(q\right)}^{X-ray}e^{iqx}dq$$

where q is a scattering vector, x is the position, and V is the volume of the unit cell.

(ii) The positions ($x_{j}{}^{\mathrm{neutron}}$) and thermal parameters ($U_{j}{}^{\mathrm{neutron}}$) of atomic nuclei derived by the WAND data analysis are read out, and the electron density distributions [$\rho {\left(x\right)}^{neutron}$] of the isolated atoms are calculated by assuming the spherical electron distributions at the nuclear positions, where the X-ray scattering factors ($f_{j}{\left(q\right)}^{X-ray}$) are utilized in the calculation of the structure factors $F_{sph}{\left(q\right)}^{neutron}$.

(5) $$\rho {\left(x\right)}^{sphere}=\left(1/V\right)\int F_{sph}{\left(q\right)}^{neutron}e^{iqx}dq$$

(6) $$F_{sph}{\left(q\right)}^{neutron}=\sum_{j}f_{j}{\left(q\right)}^{X-ray}e^{-iqx_{j}{}^{\mathrm{neutron}}}e^{-iqU_{j}{}^{\mathrm{neutron}}}$$

(iii) The subtraction Δρ(x) between the total electron distribution [$\rho {\left(x\right)}^{X-ray}$] and the hypothetic spherical electron distribution [$\rho {\left(x\right)}^{sphere}$] is calculated as

(7) $$\Delta \rho \left(x\right)=\rho {\left(x\right)}^{X-ray}-\rho {\left(x\right)}^{sphere}=\left(1/V\right)\int \left[F_{obs}{\left(q\right)}^{X-ray}-F_{sph}{\left(q\right)}^{neutron}\right]e^{iqx}dq$$

**Figure 27:**
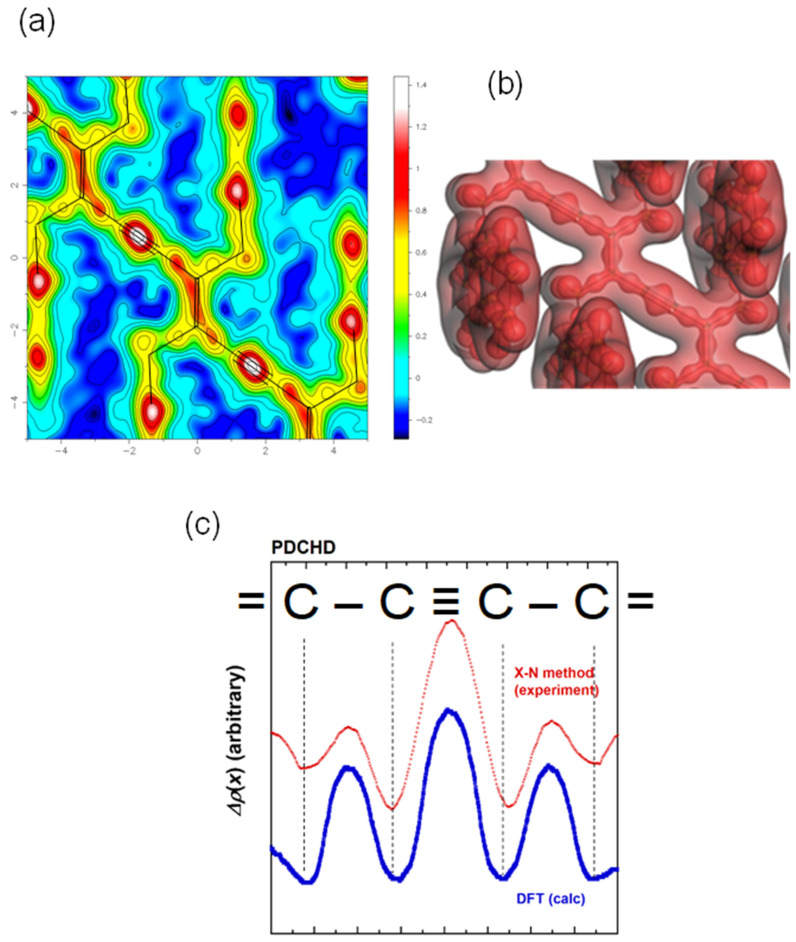
(**a**) The bonded electron density map obtained for PDCHD crystal by the X-N method. (**b**) The 3D map of electron density distribution calculated by the density functional theory. The pale red indicates the total electron density distribution, and the dark red indicates the bonded-electron density [42]. The DFT calculation was performed using a DMol^3^ program of Materials Studio package (Biovia-Accelrys). The GGA-PBE functional and DNP basis set were used. The details were described in the reference [42]. (**c**) The comparison of the bonded electron density distribution along the skeletal chain between the observed result (red) and the DFT-calculated result (blue).

**Figure 28:**
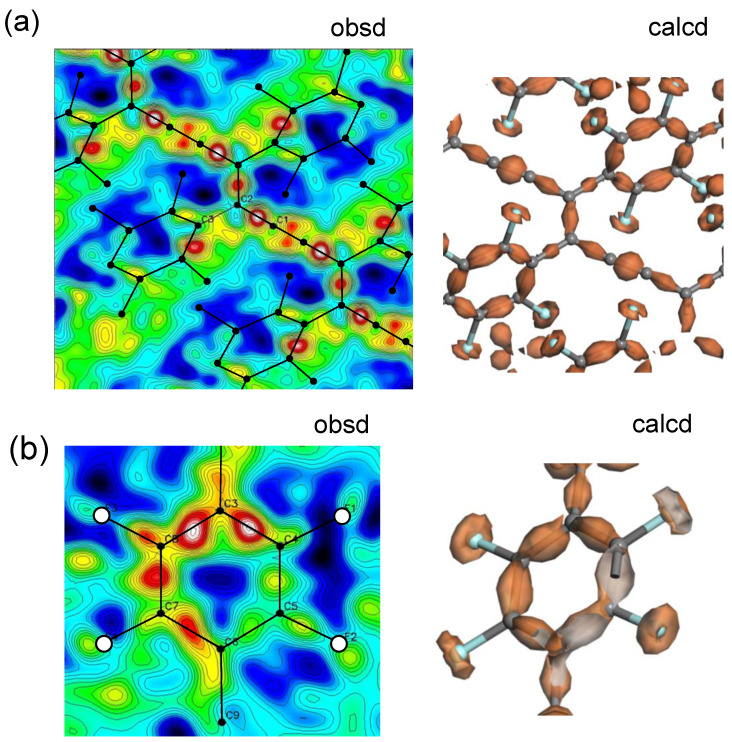
The 2D maps showing the deformed electron density distribution derived experimentally by the X-N method: (**a**) along the polydiacetylene chain (poly(FDCA) and (**b**) the tetrafluorobenzene ring part. These results are consistent with the calculated results by the density functional theory (right side), where the isosurfaces are shown by brown color.

This treatment is called the X-N method. The actually performed result is shown in Figure 27 and Figure 28, respectively, for poly(DCHD) [42] and poly(FDAC). In the case of poly(DCHD) (Figure 27a), the high electron densities are detected clearly at the centers of the C≡C, C=C, and C-C bonds. As shown in Figure 27c, the thus-experimentally derived bonded electron density distribution is in good agreement with the quantum-mechanically calculated result (Figure 27b), where the distribution is presented along the skeletal bonds. Figure 28 shows the result obtained for poly(FDAC). The distribution around C≡C bonds is slightly distorted, compared with poly(DCHD). This suggests the lower accuracy of the structure analysis of poly(FDAC) using the WAND data, probably since the alkyl chain parts are not deuterated and they are thermally activated at room temperature. A slight deviation of the atomic position affects the deformed electron density distribution sensitively, as explained in the paper [42]. In the benzene ring parts, the clear distribution is detected at the C=C bond centers. The electron density of the F atoms is not only on the centers of the C-F bonds, but it is also located slightly on the outer side of the F atoms because of the higher electron-withdrawing effect, as checked by the DFT calculation.

**Figure 29:**
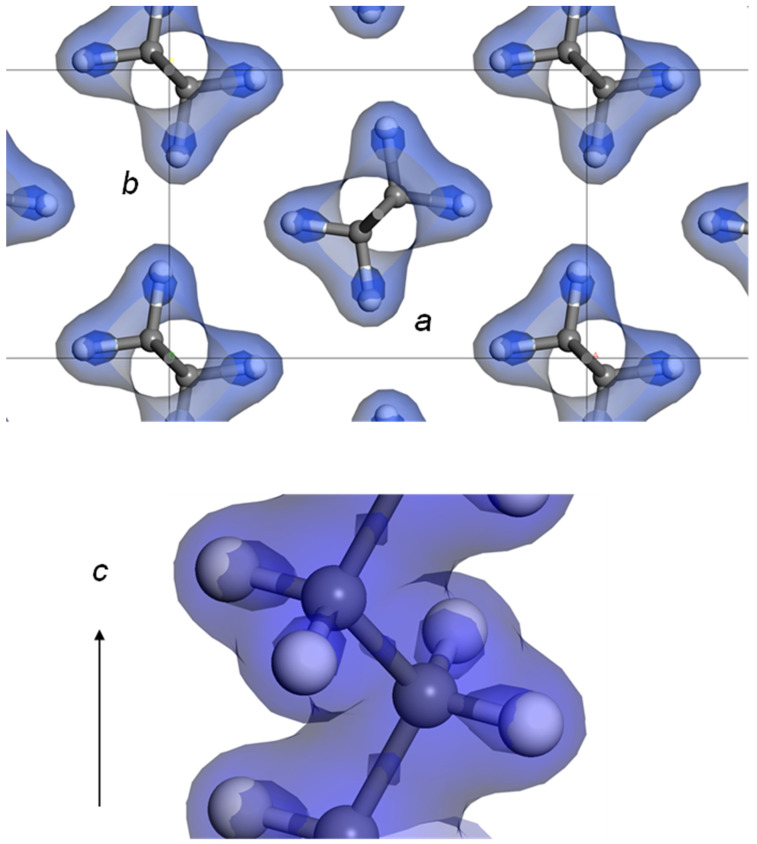
The isosurfaces of the total electron density distribution (large pale blue tubes) and bonded electron density distribution (inner dark blue lobes) predicted for the orthorhombic polyethylene crystal, which was calculated with the density functional theory.

In principle, the X-N method can be applied to the general synthetic polymers for the purpose to clarify the bonded electron distributions. For example, the bonded electron density distribution is predicted for orthorhombic polyethylene as shown in Figure 29, which was calculated with the density functional theory. The bonded electrons between the C and H atoms do not locate at the center positions between them, but the electrons are attracted toward the H atoms. In order to check such a quantum-mechanical prediction from the experimental viewpoint, the application of the X-N method may be the best way. Unfortunately, however, the accuracy of the atomic positions is still lower even for the orthorhombic polyethylene compared with the giant single crystal of polydiacetylene, making us to hesitate to challenge. These studies should be useful for the new development of polymer science in the quantum-mechanical level.

## 7. Temperature-Dependent WAND Measurements

The thermal motions of atoms are important for the study of the temperature dependence of the physical property. In some cases, the thermally induced phase transitions may occur. The WAXD method is powerful for tracing the structural changes as a function of temperature, as reported in very many papers [5]. Contrarily, the papers on WAND data collection as a function of temperature are quite limited for polymer samples. Among the many merits, the temperature-dependent WAND data are useful for detecting the thermal motion of hydrogen (deuterium) atoms in the crystal lattice. We have challenged to develop the heating/cooling systems for measuring the 2D-WAND data in a wide temperature range, which were installed in the BIX-3 and iBIX systems.

### 7.1. Crystal Structure of Orthorhombic Polyethylene at Low Temperature

**Figure 30:**
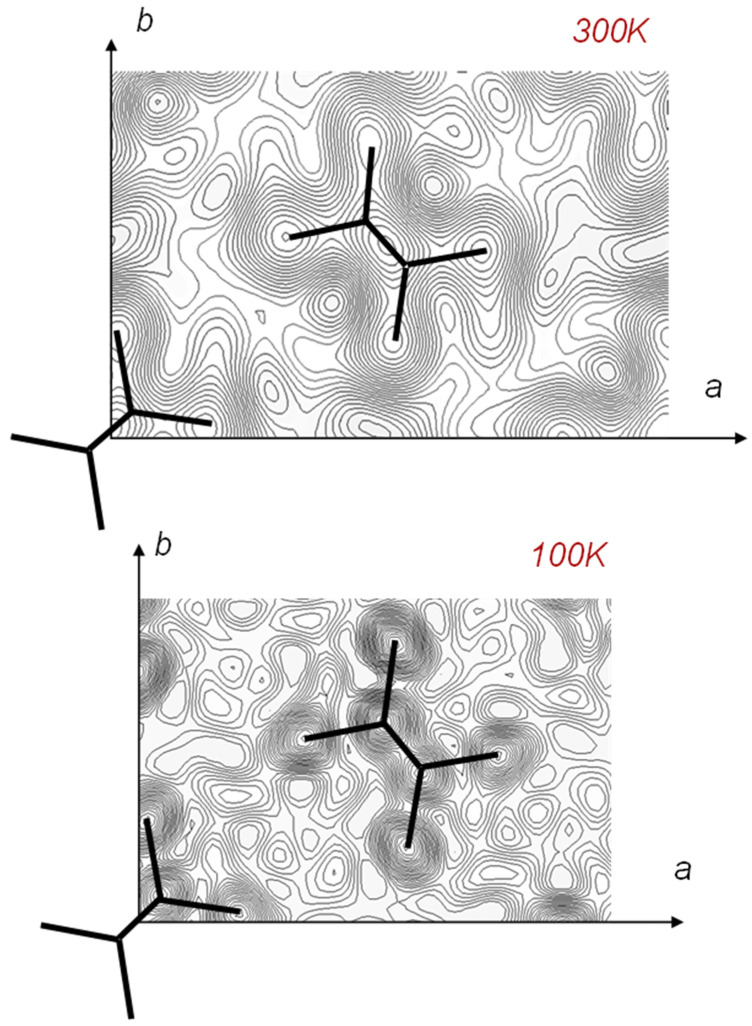
Crystal structure of orthorhombic polyethylene at 300 K and 100 K, analyzed by the 2D-WAND data analysis (BIX3). (300 K) *a* = 7.417 Å, b = 4.945 Å, *c* (chain axis) = 2.550 Å. The space group *Pnam,* and thermal parameters, C: *U*_11_ = 0.062 Å, *U*_22_ = 0.075 Å, D_1_: *U*_11_ = 0.048 Å, *U*_22_ = 0.107 Å, D_2_: *U*_11_ = 0.115 Å, *U*_22_ = 0.032 Å (100 K) *a* = 7.193 Å, b = 4.905 Å, *c* (chain axis) = 2.547 Å. *Pnam,* thermal parameters, C: *U*_11_ = 0.043 Å, *U*_22_ = 0.021 Å, D_1_: *U*_11_ = 0.027 Å, *U*_22_ = 0.074 Å, D_2_: *U*_11_ = 0.035 Å, *U*_22_ = 0.035 Å.

In Figure 4, the 2D WAND patterns of the uniaxially oriented orthorhombic HDPE-d4 sample are compared between room temperature and 100 K, where the data were collected using a cryostat installed in the BIX-3 system [48]. The diffraction peaks in the higher angle region became stronger and sharper at 100 K because of the cease of thermal motion of the zigzag chains. The Fourier maps obtained by the diffraction data analyses are shown in Figure 30. The position of the D atoms was detected more clearly at 100 K. This is consistent with the results deduced from the synchrotron X-ray diffraction data analysis made at the low temperatures [49]. Similar results were obtained by the analysis of the iBIX data.

### 7.2. Temperature Dependence of WAND Data in the Heating Process

**Figure 31:**
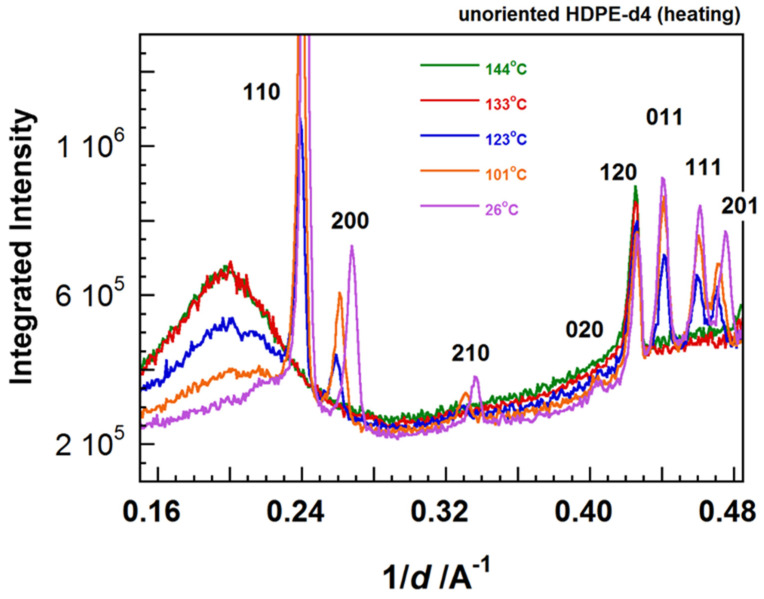
Temperature dependence of the TOF-WAND profiles measured for the unoriented HDPE-d4 sample in the heating process, where the homemade heater was installed in the iBIX system.

The heating system was produced by us and installed in the iBIX system. The sample was put in a cell sandwiched by a pair of heaters. The geometrical constraints were serious in the setting of the heater in the narrow iBIX system. As already mentioned, however, the TOF method made it possible to do so because the sample did not need to rotate. Figure 31 shows the 1D WAND profiles measured for the unoriented HDPE-d4 sample in the heating process. The diffraction peaks shifted toward the lower angle side due to the thermal expansion of the unit cell. These crystalline peaks disappeared totally above the melting point, and the amorphous halo peak increased the intensity remarkably. The detailed analysis is now given.

## 8. WAND Measurements under the Tensile Force

**Figure 32:**
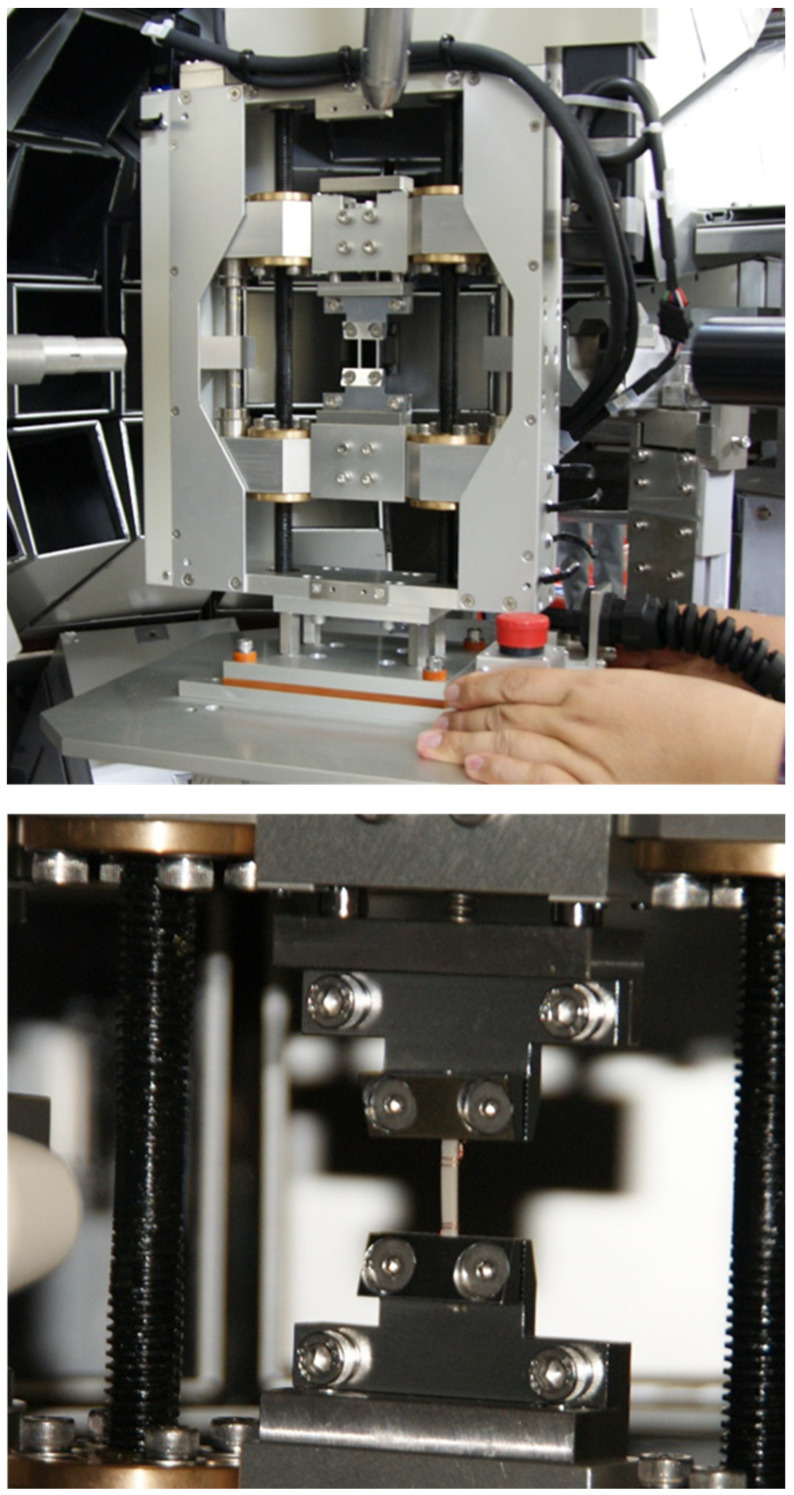
A stretching instrument produced for the tensile experiment in the iBIX system, J-PARC. A bundle of the oriented rods was set vertically.

**Figure 33:**
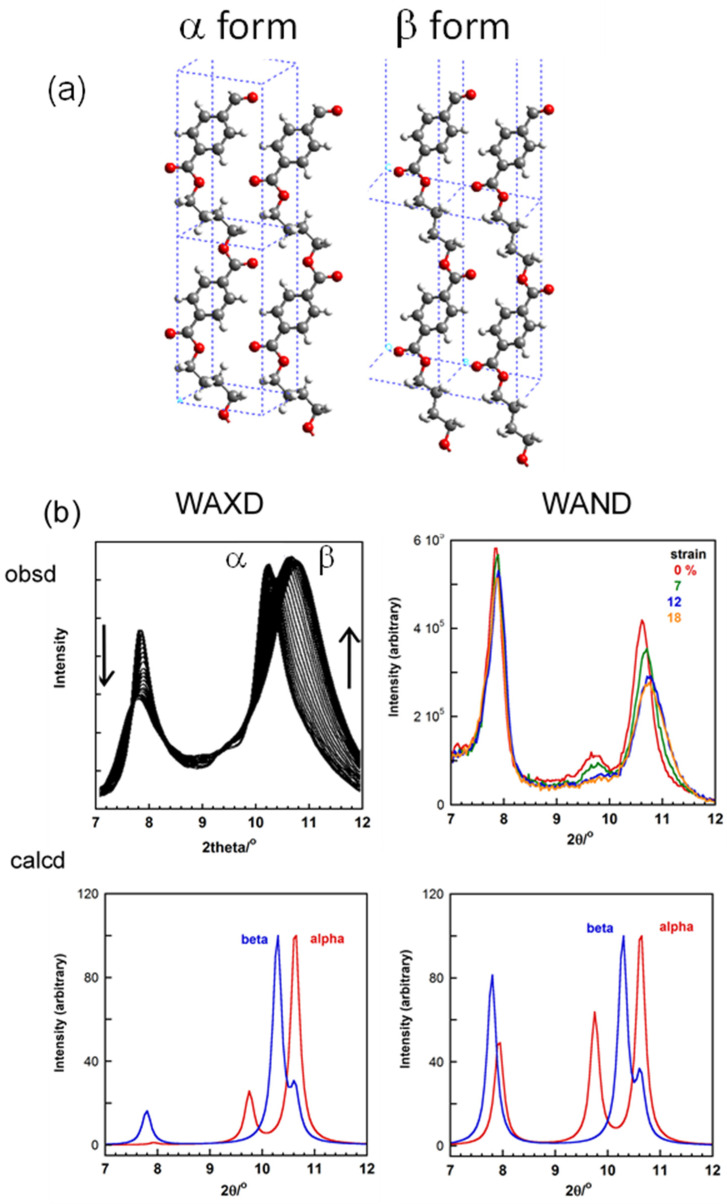
Stress-induced phase transition of a uniaxially oriented PTMT sample. (**a**) The crystal structure of the α and β forms. (**b**) The strain dependence of the WAXD and WAND equatorial line profiles in comparison between the observed and calculated profiles.

Some polymers show the phase transition by the application of a tensile force. The typical example is seen for the uniaxially oriented poly(tetramethylene terephthalate) (PTMT) sample [50,51,52]. The stable crystal phase under tension free condition is the α form, which takes the skeletal chain conformation of TGT$\bar{G}$T with the contracted repeating period. The application of a tensile stress beyond the critical value causes the discontinuous transition to the β form of the fully extended all-trans chain conformation. The structures of these two crystalline forms were analyzed by the several groups based on the X-ray diffraction data analyses [53,54,55]. However, the thus-proposed local conformations of the O(CH_2_)_4_O segmental part have not yet been established well, as seen in the doubtful interpretation of the IR (and Raman) spectral data [51,52]. In order to check these ambiguities, the PTMT samples with the deuterated methylene segments were synthesized, and the WAND data of the oriented samples were measured under the tensile force. The snapshot of the stretching device specialized for the WAND experiment is shown in Figure 32. The experimental data are shown in Figure 33 in comparison between the WAXD and WAND data. With an increase of the tensile strain, the equatorial line profiles of the α and β forms changed. However, the relative intensity and peak positions are not very well reproduced in the calculation. This might be originated from the still ambiguous crystal structure models. The quantitative analysis is now being performed.

## 9. Summary and Future Perspectives

In this review, we introduced our recent studies about the crystal structure analyses of synthetic polymers by the hybrid combination of WAXD and WAND techniques. Because of the limited number of the broad and (sometimes diffuse) diffraction peaks, the highly reliable crystal structure analysis of polymer substances is quite difficult to perform. The utilization of a high-energy synchrotron X-ray beam has allowed us to collect a greater number of the observable diffractions, which is helpful for the accurate structure analysis. In fact, the structure analyses of such representative multi-purpose polymers as PE, POM, PLLA etc. were carried out satisfactorily, by which the mechanical properties were predicted theoretically with the highest reliability. The WAND data collected for the deuterated species of these polymer species allowed us to confirm the reliability of the crystal structures derived by the X-ray data analyses. However, in some cases, the crystal structure model derived by the X-ray data analysis could not reproduce the observed WAND data at all. The typical examples were presented here for at-PVA and its iodine complex. In the latter case, the remarkable difference of the X-ray and neutron scattering powers between the various atomic groups (C, D, O) and (K and I) gave inconsistent results of the structure model. The X-ray data are governed mainly by the (K and I) atomic groups, even if the positions of (C, D (H), O) groups are not correct. On the other hand, the WAND data are determined mainly by the (C, D, O) groups. The final structure model was obtained successfully by introducing the statistical disorder of the chain packing, and both of the WAXD and WAND data were consistently reproduced well. In addition, the hybrid usage of WAXD and WAND data helps us to evaluate the bonded electron density distribution along the polymer chain by the so-called X-N method. The quantitative agreement was obtained between the thus-experimentally-derived result and the quantum-mechanical prediction. However, the successful results have been obtained only for quite limited polymer species, the giant single crystals of polydiacetylene. It should be a good challengeable theme to apply the X-N method to such general polymer substances as polyethylene, polyoxymethylene and so on. Another challenge is to trace the time-resolved change of crystal structure in the solid-state polymerization reaction. By collecting the WAXD and WAND data at high time resolution, the concrete information of the bonded electron density distribution change may be obtained as a function of time. As mentioned in the present review, we challenged to trace the structural evolution in the solid-state polymerization reaction of diacetylene single crystals (FDAC). The approximate description of the photo-induced polymerization reaction of FDAC was given in this article. However, it is still not satisfactory enough to reveal deeper information of the reaction.

As presented in the last section, the hybrid usage of WAXD and WAND techniques is needed for the study of the structural changes in the phase transitions under such an externally applied field as temperature, stress, or electric field [56,57]. The instruments for the WAXD data collection have been developed already, while those of WAND experiment are still not matured. The several reasons are listed: the weak neutron source, the necessity to use a deuterated sample of a large volume, etc. The further development of the neutron facility, for example, J-PARC system, may be helpful for the solution of these problems.

**Figure 34:**
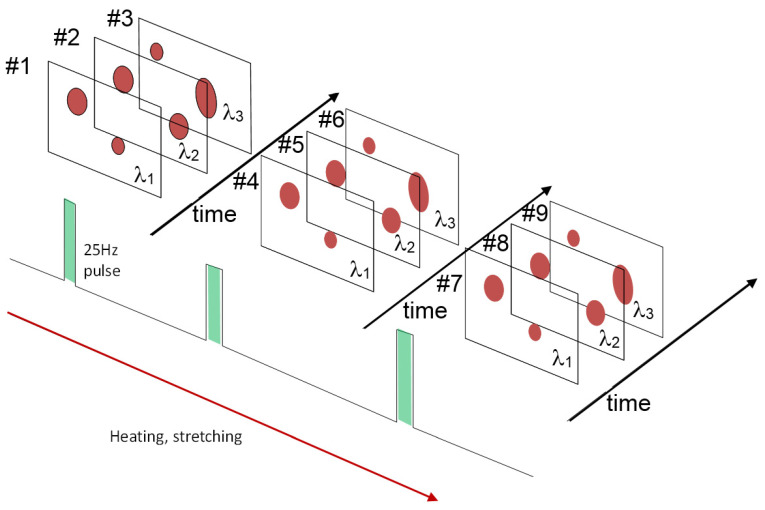
A series of TOF data. If the phase transition occurs during the TOF measurement, all the snapshots are those at the different timings. The structural changes can be traced at a quite high time resolution in principle. (Of course, the effect of wavelength on the patterns must be taken into consideration in the analysis).

The above-mentioned J-PARC is characterized as a TOF system, which may be useful for the further development of neutron science. As mentioned, the neutron beams generated by the spallation technique are pulses, which consist of the neutron components of the widely distributed wavelengths. In the J-PARC system, a series of these neutron pulses are generated at 25 Hz, and they arrive at the sample position at every 40 msec [58,59]. The scattered neutron-beam components of the different wavelengths enter the various positions (*x*, *y*) of the 2D detector at the different timing (*t*), and so we have such a series of data as illustrated in Figure 34. All of these data give us the different structure information in principle. It might be possible to build up many types of experiments by arranging the concrete processes of the data collection [60].

As pointed out above, the structural analysis based on the WAND data has not yet been reported in many papers. However, once when the experimental techniques and the data analytical methods are developed further, we may obtain many useful structure information, for example, the information about the mechanically or thermally induced changes of the positions and thermal mobility of the hydrogen (deuterium) atoms in the crystal lattice. This information is indispensable for the quantitative discussion of the structure-property relation, since the intermolecular interactions between the non-bonded H…H pairs govern the physical property and its anisotropy quite sensitively.

The usage of powerful synchrotron X-ray source has accelerated the development of the structural science of the polymer materials. The complementary combination of WAXD and WAND techniques will accelerate the developing speed of this research field more remarkably.

In the present review, we focused only on the wide-angle X-ray and neutron scatterings. In order to discuss the structure–property relation of synthetic polymers, of course, we need to combine the wide-angle and small-angle scattering data. This can be said not only for the X-ray scattering but also for the neutron scattering. The concrete research is referred to in the literature [5].

## Figures and Tables

**Table 1:** X-ray and neutron scattering powers of the various atomic species.

Atomic Species	H	D	C	O	K^+^	I^−^
Number of electrons	1	1	6	8	18	54
Coherent neutron scattering amplitude (10^−13^ cm)	−3.74	6.67	6.65	5.80	3.67	5.28
Coherent neutron cross-sectional area (10^−24^ cm^2^)	1.76	5.59	5.55	4.23	1.69	3.50
Incoherent neutron cross-sectional area (10^−24^ cm^2^)	80.27	2.05	0.00	0.00	0.27	0.31
X-ray scattering factor at 2θ = 0°	1	1	6	8	18	54
